# PorV factor of the type IX secretion system and PosF porin act as adhesins in *Riemerella anatipestifer* infection

**DOI:** 10.1186/s13567-025-01550-8

**Published:** 2025-06-08

**Authors:** Sen Li, Yanhua Wang, Congran Ning, Rongkun Yang, Yaxin Wu, Xu Cheng, Keke Ren, Minghua Huang, Xiong Liu, Naiji Zhou, Wanpo Zhang, Sishun Hu, Yuncai Xiao, Zili Li, Hongbo Zhou, Zhengfei Liu, Zutao Zhou

**Affiliations:** 1 College of Veterinary Medicine, Huazhong Agricultural University, Wuhan, China; 2 State Key Laboratory of Agricultural Microbiology, Huazhong Agricultural University, Wuhan, China; 3 Key Laboratory of Preventive Veterinary Medicine in Hubei Province, Huazhong Agricultural University, Wuhan, China; 4 Hubei Hongshan Laboratory, Huazhong Agricultural University, Wuhan, China

**Keywords:** *Riemerella anatipestifer*, DEF cells, adhesion, outer membrane proteins, virulence, T9SS, porin protein, microbial‒host interactions

## Abstract

*Riemerella anatipestifer* infection is a critical disease that is a major threat to the poultry industry worldwide. The adhesion and invasion of host cells are key steps in the primary stages of bacterial infection. However, the outer membrane proteins that mediate these events in *R. anatipestifer* are poorly characterized. In this study, the PorV and PosF proteins, as well as the previously described OMP71 protein, were identified as important mediators of the adhesion and invasion of duck embryo fibroblast (DEF) cells by *R. anatipestifer*. Affinity chromatography-based surface proteomics was used to screen for adhesion proteins. The surface proteins on DEF cells were labelled with biotin-avidin to enrich for outer membrane proteins of *R. anatipestifer,* which generated 11 candidate proteins that were tested further. Protein adhesion and blocking assays and polyclonal antiserum inhibition analysis revealed that the PorV, PosF, and OMP71 proteins are adhesion factors. Knockout of *porV* or *posF* reduced the adhesion and invasion of *R. anatipestifer* in DEF cells. Moreover, the pathogenicity of the mutant strains was significantly attenuated, which supports the hypothesis that PorV and PosF are important virulence factors required for the pathogenicity of *R. anatipestifer*. The PorV protein is a key component of the type IX secretory system and is responsible for transporting effector substrates to the extracellular environment, whereas PosF belongs to the porin superfamily of barrel-shaped transmembrane proteins. This is the first description that PorV is an adhesin involved in host‒microbial interactions, which represents a breakthrough in pathogenicity studies of *R. anatipestifer* and other members of *Flavobacteriaceae*.

**Supplementary Information:**

The online version contains supplementary material available at 10.1186/s13567-025-01550-8.

## Introduction

*Riemerella anatipestifer* is a member of the *Flavobacteriaceae* family and causes infectious serositis in poultry, including waterfowl, chicken, and turkey [1]. At least 21 serotypes of *R. anatipestifer* have been reported worldwide, with serotypes 1, 2, 6, and 10 being the most prevalent in China [2, 3]. Septicemia caused by *R. anatipestifer* infection leads to significant economic losses in the waterfowl industry [4]. Moreover, *R. anatipestifer* has also been identified in breeding hens and commercial chickens and, as one of the most severe bacterial pathogens in poultry [5], is exerting an increasing impact on the poultry industry worldwide [6, 7]. The weakness of cross-protection among serotypes greatly reduces the immune effects of current vaccines [8, 9]. Virulence factors, including outer membrane proteins (OMPs), lipopolysaccharides, capsular polysaccharides, two-component systems, and the type IX secretory system (T9SS), are implicated in the pathogenicity of *R. anatipestifer* [10–15]. However, the detailed pathogenic mechanisms that underpin *R. anatipestifer* infection require urgent clarification.

Gram-negative bacteria possess a complex cell envelope that comprises an outer membrane (OM), a peptidoglycan layer, and an inner membrane. The OM is a critical interface for host‒pathogen interactions, with OMPs playing pivotal roles in adhesion, colonization, and immune evasion [16]. These proteins, often organized as β-barrel structures, facilitate bacterial attachment to host cells, which is a prerequisite for infection. Adhesion and invasion mediated by OMPs are fundamental events in the interaction between Gram-negative bacterial pathogens and host cells [17–19], including the 43 K OMP of *Fusobacterium necrophorum*, Pla of *Yersinia pestis*, OmpV of *Salmonella typhimurium*, Omp33 of *Acinetobacter baumannii*, and Opa proteins of *Neisseria* spp. [20–24]. *R. anatipestifer* is detectable in the upper respiratory tract of clinically healthy ducklings and has the ability to cross the blood‒brain barrier [25–28]*.* These abilities are dependent on the interaction between *R. anatipestifer* adhesins and the receptors of host cells, which highlights that adhesion is a key step in the pathogenesis of *R. anatipestifer*. *R. anatipestifer* deploys adhesins to attach to and invade host cells and then spreads in the blood, which promotes increased vascular permeability and leads to fibrous pericarditis, perihepatic inflammation, meningitis, and ballonets in infected animals. The OmpH, OMP76, OmpA, and GldM proteins of *R. anatipestifer* are implicated in virulence, potentially as adhesins [29–32]. However, the exact identity of the adhesins that mediate interactions between *R. anatipestifer* and host cells has not been elucidated.

The T9SS exists only in *Bacteroidetes,* in which the system mediates cellular movement, protein secretion, and pathogenicity [33, 34]. Most of the proteins secreted by the T9SS harbor an N-terminal signal peptide and a C-terminal conserved domain (CTD). These proteins are synthesized in the cytoplasm in the form of polyproteins, which are transported across the intima to the periplasmic space via the Sec system. The T9SS complex recognizes the CTD domain, transports the protein to the OM, and then secretes it to the bacterial surface, which is critical for systemic effects, including pathological injury, tissue necrosis, and host immune escape [35, 36]. In previous studies, we demonstrated that *R. anatipestifer* RA-YM possesses a complete T9SS and that the SprA and SprT components, as well as the secreted serine protease SspA, are virulence factors [15, 37, 38].

Thus, this study aimed to screen for and identify adhesins that contribute to the attachment of *R. anatipestifer* to host cells and to explore their role in pathogenesis. The surface proteins of duck embryo fibroblast (DEF) cells were labelled via a biotin-avidin system, and the interactions of the OMPs of *R. anatipestifer* RA-YM were detected. The adhesin identities of PorV and PosF were confirmed among the candidate OMPs and validated further using protein direct adhesion assays, competitive inhibition experiments, antibody blocking assays, and gene knockouts. Importantly, the PorV and PosF factors also contributed to the virulence of *R. anatipestifer*. Therefore, our findings help elucidate the pathogenic mechanisms that underpin host‒microbial interactions in *R. anatipestifer* and suggest that PorV and PosF may form the basis of novel subunit vaccines.

## Materials and methods

### Ethics statement

Animal experiments were performed in accordance with the recommendations for the Care and Use of Laboratory Animals from the Research Ethics Committee, Huazhong Agricultural University, Hubei, China (approval no. HZAURAB-2024-0011). The procedures used in the studies involving animals were in accordance with the ethical standards of the institution or practice at which the studies were conducted.

### Bacteria, plasmids, and growth conditions

The bacterial strains, plasmids and primers used in this study are listed in Additional file 1. DEF cells were purchased from the American Type Culture Collection. *R. anatipestifer* RA-YM was grown on TSA agar plates or in TSB liquid media (Becton, Dickinson and Company, Franklin Lakes, USA) at 37 °C with 5% CO_2_. Newborn bovine serum was purchased from EVERY GREEN, Zhejiang Tianhang Biotechnology (Huzhou, China). *Escherichia coli* DH5α was used for plasmid propagation, and *E. coli* BL21 (DE3) (Weidi, Shanghai, China) was used for protein expression. *E. coli* X7213 is auxotrophic for diaminopimelic acid (100 μg/mL; Sigma‒Aldrich, Burlington, USA) and was used for conjugative transfer between *E. coli* and *R. anatipestifer*. *E. coli* strains were subsequently grown in lysogeny broth (LB) and LB agar at 37 °C. The concentrations of antibiotics used (Sigma‒Aldrich) were as follows (μg/mL): ampicillin, 50%; spectinomycin, 100%; and erythromycin, 4.

### Screening of surface adhesion molecules of *Riemerella anatipestifer* RA-YM

DEF cells were grown in Ham’s F12 Nutrient Mixture (Sigma‒Aldrich) with 10% fetal bovine serum (Gibco, Carlsbad, USA) containing penicillin‒streptomycin (1%; Gibco) at 37 °C with 5% CO_2_ for 48 h to generate a dense monolayer. The cells were washed three times with ice-cold PBS (pH 8.0). The surface molecules of DEF cells were labelled with EZ-Link NHS-PEG4-Biotin (2 mM; Thermo Fisher, Waltham, USA) on ice for 30 min (experimental group). The control cells were incubated in parallel with ice-cold PBS (pH 8.0) (control group). The cells were subsequently washed with glycine (100 mM), scraped into tubes, and washed with PBS (1 mL) in triplicate to remove unbound biotin. PBS (1 mL) containing Zwittergent 3–14 (2%; MCE, Monmouth Junction, USA) was added to each tube at 4 °C for 30 min for surface molecule extraction. The sample was centrifuged at 13 000 × *g* for 30 min, and the supernatant was transferred to fresh tubes and incubated with Pierce® Monomeric Avidin Agarose (Thermo Fisher) for purification of biotinylated DEF cell surface proteins. The agarose was subsequently washed with PBS, and OMPs (100 μg) of *R. anatipestifer* RA-YM were added. The negative control group included OMPs incubated with agarose alone. OMPs of *R. anatipestifer* RA-YM were extracted and separated based on methods described previously [38]. After incubation for four hours, agarose-bound proteins were eluted with elution buffer (2 mM D-biotin in PBS, pH 8.0) for subsequent LC‒MS/MS sequencing (Novegene, Beijing, China).

### Cloning, expression, and purification of His_10_-OMPs and antibody production

Candidate OMP genes lacking the signal peptide sequence were cloned and inserted into pET-16b, which possesses a 10 × His tag, via the restriction enzyme *Nde*I (Thermo Fisher) and the 2 × MultiF SeamLess Assembly Mix (ABclonal, Wuhan, China). The recombinant plasmids were subsequently transformed into *E. coli* BL21 (DE3) for gene overexpression and protein purification. Antibody production was described previously [30]. Briefly, purified His_10_-OMPs (500 μg) were mixed with fully emulsified adjuvant and used to immunize Japanese big ear white rabbits on days 14, 28, and 42. Serum was collected and stored at −80 °C.

### Immunofluorescence assay

Confluent monolayers of DEF cells were infected with *R. anatipestifer* RA-YM (MOI = 100), which was labelled with 10 μmol carboxyfluorescein diacetate succinimidyl ester (cFDA-SE, MCE). The control group was infected with *R. anatipestifer* RA-YM not labelled with cFDA-SE. The cells were incubated at 37 °C for 2 h and then washed three times with sterile PBS. The cells were examined with an IX73 inverted fluorescence microscope (Olympus, Hachioji, Japan). For assays of biotin-labelled DEF cells, confluent monolayers were fixed with 4% paraformaldehyde for 30 min at room temperature. Two hundred micromoles of EZ-Link NHS-PEG4-Biotin were added, and the mixture was incubated for 30 min at 37 °C. The samples were washed three times with PBS, followed by incubation with FITC-streptavidin (1:500 dilution) (BioLegend, San Diego, USA) for 30 min at 37 °C. The cells were again washed in triplicate with PBS, and the nuclei were stained with DAPI (Sigma‒Aldrich). After a final wash, the cells were examined by fluorescence microscopy. For the adhesion assays of the OMPs of *R. anatipestifer* RA-YM, DEF cells were fixed with 4% paraformaldehyde for 30 min at room temperature and then incubated with 100 μg of recombinant His_10_-OMPs at 4 °C overnight. The cells were washed three times with PBS and incubated sequentially with a mouse anti-His mAb (1:100 dilution) (ABclone) and a FITC-conjugated goat anti-mouse IgG antibody (1:100 dilution) (ABclone). The cells were then washed three times with PBS and examined by fluorescence microscopy.

### Western blotting

*R. anatipestifer* RA-YM (10^9^ CFU) was collected and resuspended in 400 μL of PBS. The cells were disrupted by ultrasonication, and 5 × SDS‒PAGE sample loading buffer (100 μL; ABclone) was added. The mixture was heated at 95 °C for 10 min and then centrifuged at 13 000 × *g* for 1 min, after which the supernatant was used for SDS‒PAGE analysis. Proteins were transferred to a PVDF membrane (Bio-Rad, Hercules, USA). The membrane was incubated in blocking buffer (5% (w/v) skim milk in TBST) for 2 h at room temperature, and rabbit-anti-OMP pAb (1:500 dilution) and HRP-conjugated goat anti-mouse IgG (H + L) (1:10 000 dilution) were added sequentially to detect the OMPs of *R. anatipestifer* RA-YM.

### Flow cytometry

*R. anatipestifer* RA-YM was grown to logarithmic phase, and the concentration was adjusted to 10^9^ CFU/mL. The cells (100 μL) were incubated with rabbit anti-OMP pAb (1:20 dilution) for 1 h at 37 °C and then washed three times with PBS containing 1% BSA. FITC-conjugated goat anti-rabbit IgG (1:100 dilution) (ABclone) was added, and the samples were incubated at 37 °C for 30 min. The cells were washed in triplicate with PBS supplemented with 1% BSA, and a CytoFLEX instrument (Beckman Coulter, Indianapolis, USA) was used to detect the surface localization of the candidate OMPs.

### Competitive inhibition assays

Protein competition and antibody blocking assays were used to assess whether candidate OMPs inhibited *R. anatipestifer* RA-YM adhesion to DEF cells. For protein competition assays, DEF cells were spread on a 24-well plate, 100 μg of OMPs were added to each well, and the mixture was incubated at 37 °C for 1 h. BSA (100 μg) was added as a negative control. The cells were washed three times with sterile PBS, *R. anatipestifer* RA-YM (5 × 10^7^ CFU; MOI = 100) was added, and incubation was continued at 37 °C for 2 h. The samples were washed three times with sterile PBS to remove nonadherent bacteria. DEF cells were lysed with 0.25% trypsin (200 μL; Gibco), followed by dilution, plating on TSA agar, incubation at 37 °C for 48 h, and colony enumeration. For the antibody blocking assays, *R. anatipestifer* RA-YM (5 × 10^7^ CFU; MOI = 100) was incubated with antibodies against OMPs (1:20 dilution) at 37 °C for 1 h. Bacteria incubated with negative control serum (1:20 dilution) were used as a control. The cells were washed in triplicate with sterile PBS, added to 24-well cell plates, and incubated at 37 °C for 2 h. The samples were washed three times with sterile PBS to remove nonadherent bacteria. DEF cells were lysed with 0.25% trypsin (200 μL), followed by dilution, plating on TSA agar, incubation at 37 °C for 48 h, and colony enumeration.

### Construction of *R. anatipestifer *RA-YM Δ*porV*, Δ*posF*, CΔ*porV*, and CΔ*posF* mutants

*R. anatipestifer* RA-YM Δ*porV* and Δ*posF* mutants were constructed according to methods described previously [30]. Briefly, the 5’ and 3’ homology arms of the *porV* and *posF* genes were amplified via PCR using the RA-YM genome as a template, and the spectinomycin resistance gene was amplified using the pIC-333 plasmid as a template. The PCR products were purified with the Cycle Pure Kit (Omega, Norcross, USA). The pRE-112 plasmid was digested with *Kpn*I and *Sph*I (Thermo Fisher) for 30 min at 37 °C. Homologous recombination occurred between the digested pRE-112 plasmid; the 5’ and 3’ homology arms of the *porV* gene or *posF* gene; and the spectinomycin resistance gene for the assembly of pRE-112-*porV*-LSR and pRE-112-*posF*-LSR using the 2 × MultiF SeamLess Assembly Mix. The pRE-112-*porV*-LSR and pRE-112-*posF*-LSR plasmids were subsequently transformed into *E. coli* X7213, followed by conjugative transfer with *R. anatipestifer* RA-YM*.* The *porV*, *posF*, and spectinomycin resistance genes were amplified by PCR for the identification of the *R. anatipestifer* RA-YM Δ*porV* and Δ*posF* strains. The methods used to construct the *R. anatipestifer* RA-YM CΔ*porV* and RA-YM CΔ*posF* complement strains were described previously [39].

### Adhesion and invasion assays

*R. anatipestifer* RA-YM, RA-YM Δ*porV*, and RA-YM Δ*posF* strains were grown in TSB media to an OD_600_ = 0.8. The cells were washed three times with sterile PBS, serially diluted, and plated on TSA agar for enumeration. For the adhesion assay, DEF cells were spread on a 24-well plate. *R. anatipestifer* strains were grown in TSB medium to OD_600_ = 0.6–0.8, washed in triplicate with Ham’s F12 nutrient mixture, and 5 × 10^7^ CFUs of the strains (MOI = 100) were added to the wells and incubated at 37 °C for 2 h. Samples were washed three times with sterile PBS to remove nonadherent bacteria, and DEF cells were lysed with 0.25% trypsin (200 μL), followed by dilution, plating on TSA agar, incubation at 37 °C for 48 h, and colony enumeration. A total of 5 × 10^7^ CFU of *R. anatipestifer* RA-YM, RA-YM Δ*porV*, or RA-YM Δ*posF* strains (MOI = 100) were labelled with cFDA-SE (10 μmol) and incubated with DEF cells for 2 h. Samples were washed three times with sterile PBS, and the fluorescence intensity was detected by fluorescence microscopy and a Spark® Multimode Microplate Reader (TECAN, Männedorf, Switzerland). For the invasion assay, *R. anatipestifer* RA-YM, RA-YM Δ*porV*, and RA-YM Δ*posF* strains (MOI = 100) were incubated with DEF cells for 2 h and washed three times with sterile PBS. Ham’s F12 Nutrient Mixture containing gentamicin (100 μg/mL) was added (1 mL), and the mixture was incubated at 37 °C for 1 h to kill the extracellular bacteria. The cells were washed three times with sterile PBS. DEF cells were lysed with 0.25% trypsin (200 μL), followed by dilution, plating on TSA agar, incubation at 37 °C for 48 h, and colony enumeration.

### Animal experiments

*R. anatipestifer* RA-YM, RA-YM Δ*porV*, and RA-YM Δ*posF* strains were grown to OD_600_ = 0.6–0.8 in TSB, harvested by centrifugation, resuspended to OD_600_ = 1 in TSB, and transferred to fresh TSB medium at a dilution of 1:500. The OD_600_ was measured every 2 h, with three repetitions for each strain. The determination of bacterial virulence was performed as described previously [38]. Briefly, strains were cultured in TSB medium to an OD_600_ of 0.6–0.8, harvested, washed three times with sterile PBS, resuspended to an OD_600_ of 1.0, and then serially diluted to 5 × 10^9^, 5 × 10^8^, 5 × 10^7^, 5 × 10^6^, and 5 × 10^5^ CFU/mL. Two hundred and sixty 10-day-old Cherry Valley ducklings that were not immunized with the infectious serositis vaccine were divided randomly into twenty-six groups. Animals were infected with *R. anatipestifer* RA-YM, RA-YM Δ*porV*, RA-YM CΔ*porV*, RA-YM Δ*posF*, or RA-YM CΔ*posF* strains or sterile PBS via the injection of 200 μL of each duckling in flippers. The animals were observed for seven days, the number of deaths was recorded, and the LD_50_ values were determined. The heart, liver, spleen, brain, and blood of ducklings infected with 10^7^ CFU of *R. anatipestifer* strains were collected for determination of tissue bacterial loads and preparation of pathological sections. Sera were collected simultaneously for detection of cytokine levels (Meimian, Yancheng, China).

### Statistical analyses

Statistical analysis was performed using GraphPad Prism version 9.0 (GraphPad, LA Jolla, USA). One-way ANOVA and two-way ANOVA were used for assay analysis. The significance level for all analyses was set as *P ≤ 0.05, **P ≤ 0.01 and ***P ≤ 0.001.

## Results

### *R. anatipestifer* RA-YM adheres to DEF cells

**Figure 1:**
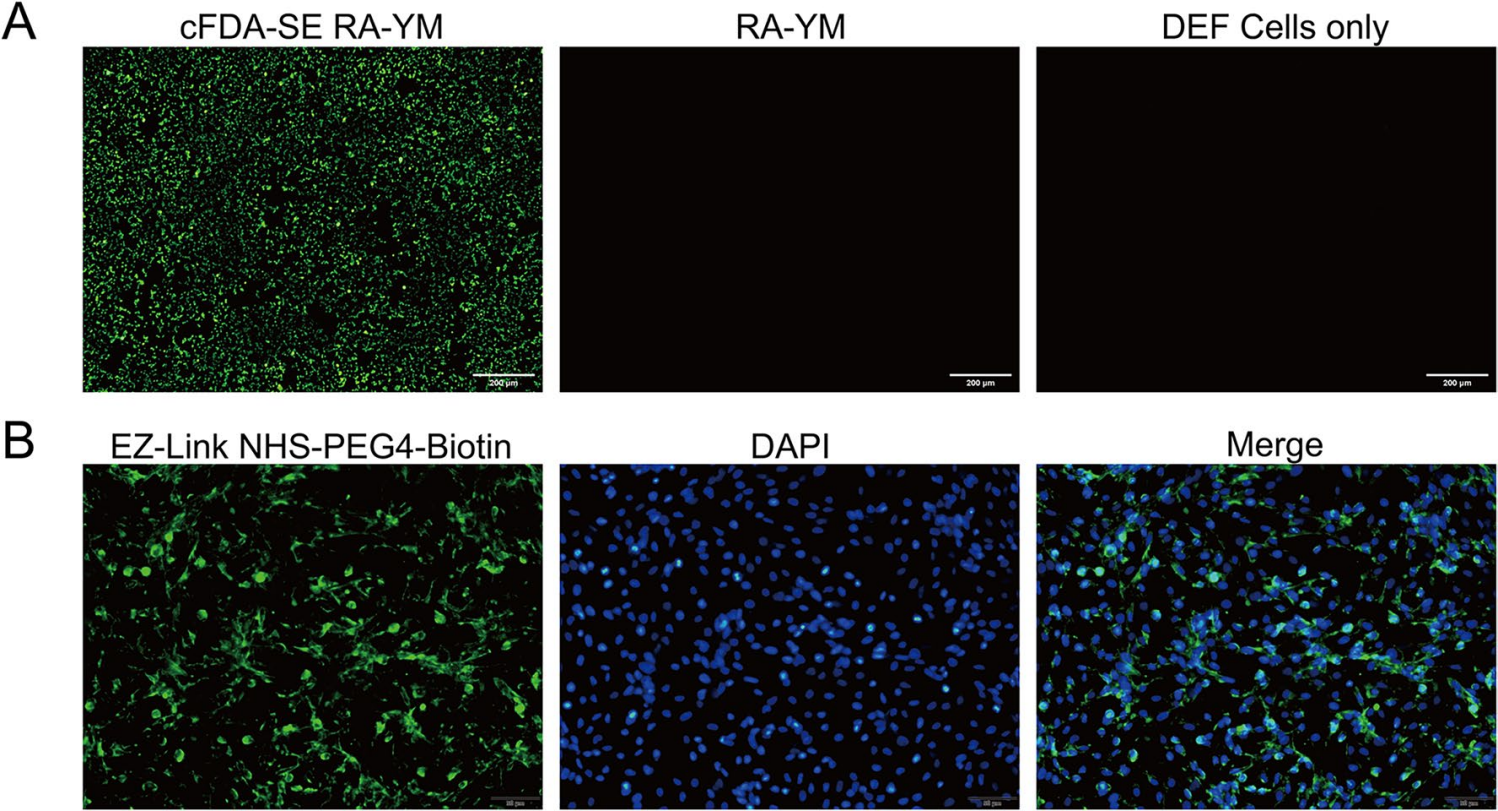
I**mmunofluorescence detection of the adhesion of* R. anatipestifer*** **RA-YM to DEF cells**. **A** Left: cFDA-SE-labelled RA-YM adheres to DEF cells. Middle: RA-YMs adhere to DEF cells. Right: DEF cells only. **B** Left: DEF cells labelled with EZ-Link NHS-PEG4-Biotin and FITC-streptavidin. Middle: nuclei of DEF cells stained with DAPI. Right: merged.

The adhesion of *R. anatipestifer* to DEF cells was reported previously [40]. This observation was investigated further by co-incubating DEF cells with *R. anatipestifer* RA-YM labelled with cFDA-SE. The fluorescently labelled strain that adhered to DEF cells exhibited fluorescence, whereas no fluorescence was observed in DEF cells alone or in those co-incubated with non-fluorescently labelled RA-YM (Figure 1A), which indicated that *R. anatipestifer* RA-YM adhered to the target cells. In addition, DEF cells were incubated with EZ-Link NHS-PEG4-Biotin-bound FITC-streptavidin, which demonstrated the labelling of surface proteins with the conjugate (Figure 1B); therefore, these proteins may be extracted by a biotin-avidin affinity system.

### Several outer membrane proteins of *R. anatipestifer* interact with surface proteins of DEF cells

**Figure 2:**
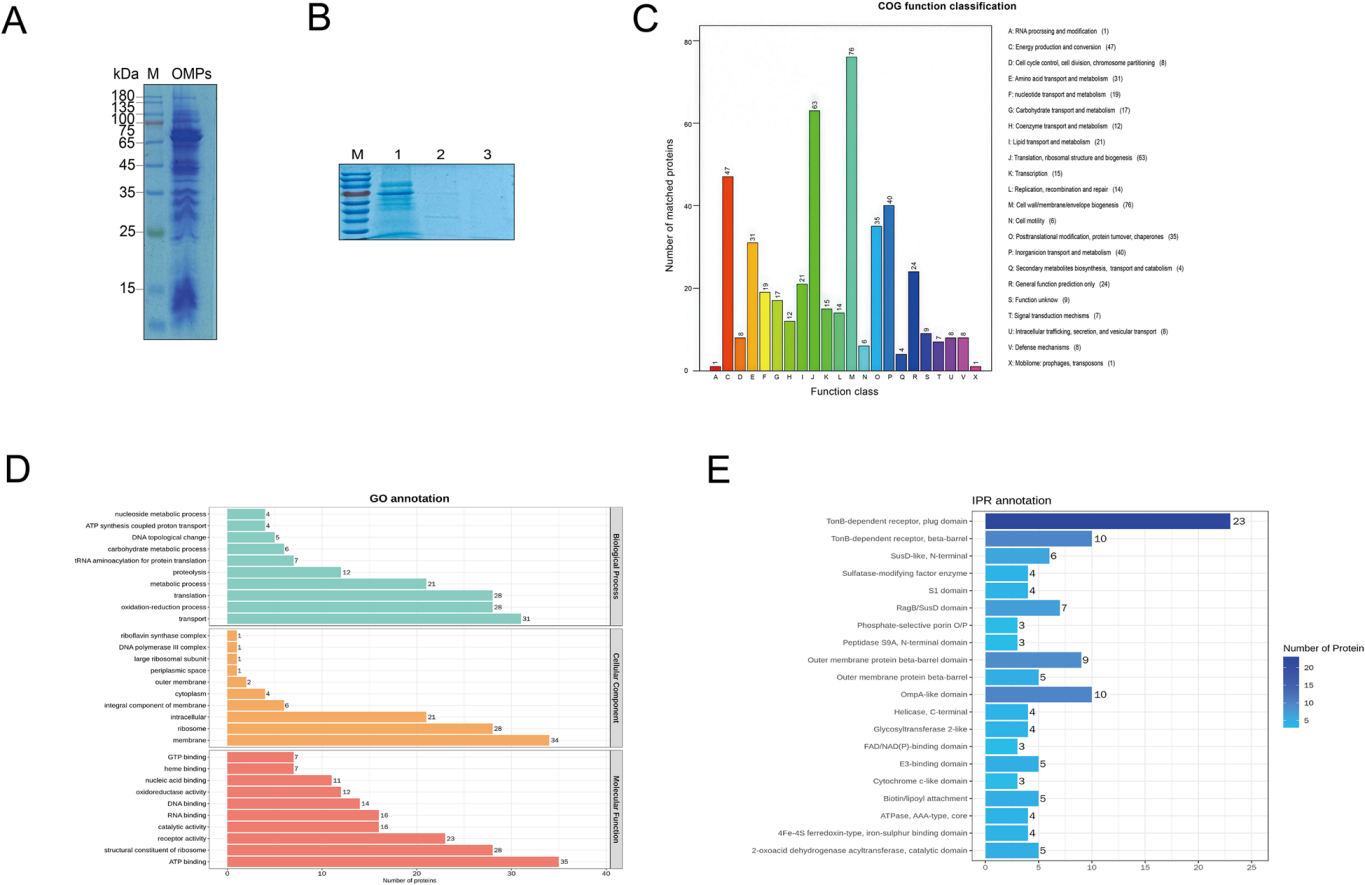
**Screening of candidate surface adhesion molecules of *R. anatipestifer* RA-YM by LC–MS/MS**. **A** Extraction of OMPs from the *R. anatipestifer* RA-YM strain by Sarkosyl. M: Protein ladder (10–180 kDa) (Mei5bio, Beijing, China) **B** Combination of OMPs of the *R. anatipestifer* RA-YM strain with the following treatments: 1, biotinylated surface proteins of DEF cells purified with monomeric avidin agarose (experimental group); 2, nonbiotinylated surface proteins of DEF cells purified with monomeric avidin agarose (control group); 3, monomeric avidin agarose without surface proteins of DEF cells (negative control group). **C** COG functional classification of candidate surface adhesion molecules. Proteins implicated in the biogenesis of the cell wall, cell membrane, and cell envelope account for the largest proportion. **D** GO annotation of candidate surface adhesion molecules. Transporters and membrane proteins account for a relatively high proportion. **E** IPR annotation of candidate surface adhesion molecules. InterProScan analysis revealed that most proteins are TonB-dependent OMP receptors.

**Figure 3:**
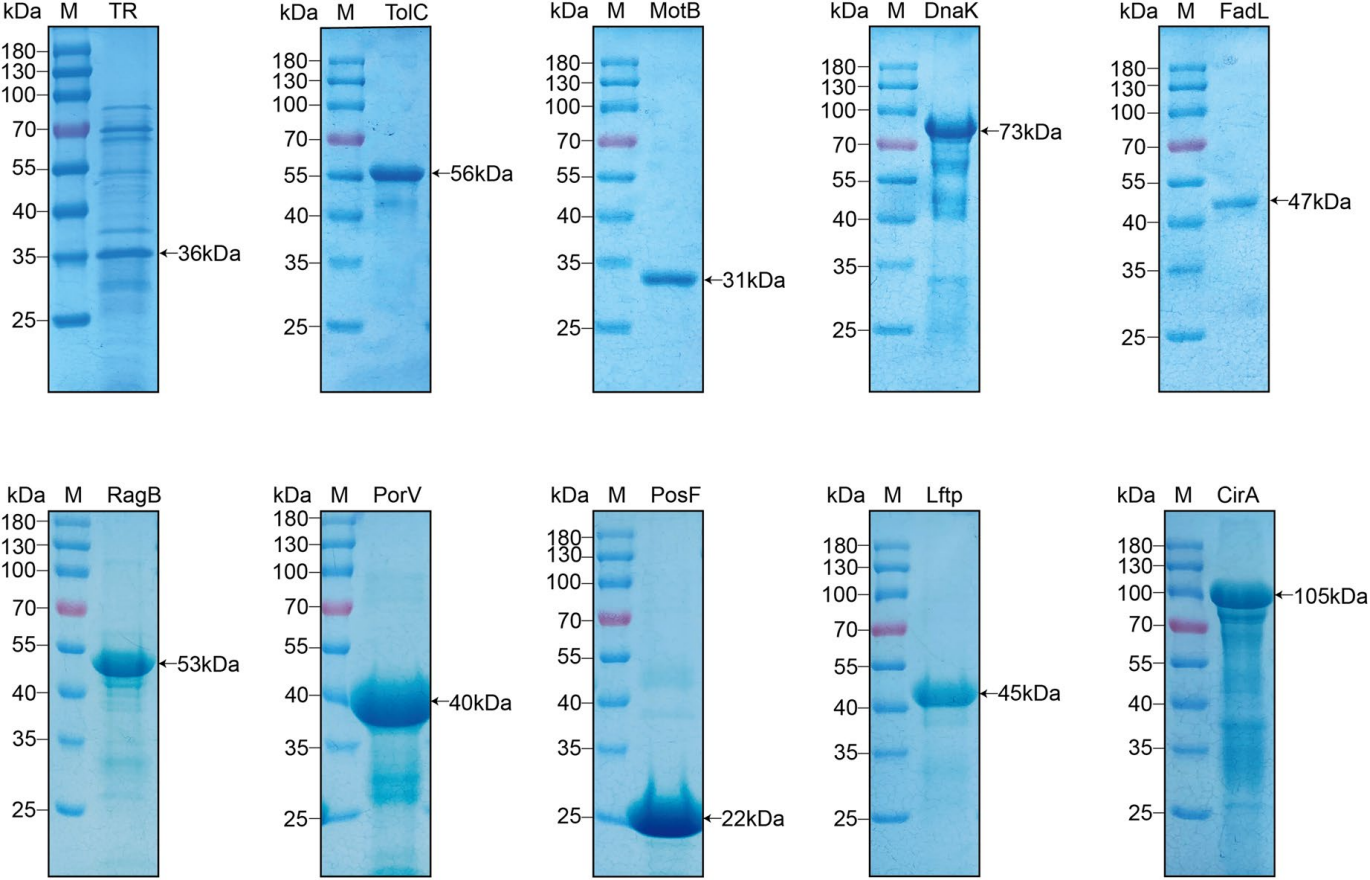
**Purified candidate adhesins (TR, TolC, MotB, DnaK, FadL, RagB, PorV, PosF, Lftp, and CirA) were analysed by SDS‒PAGE (M: PageRuler 10–180 kDa marker; Thermo Fisher)**.

**Figure 4:**
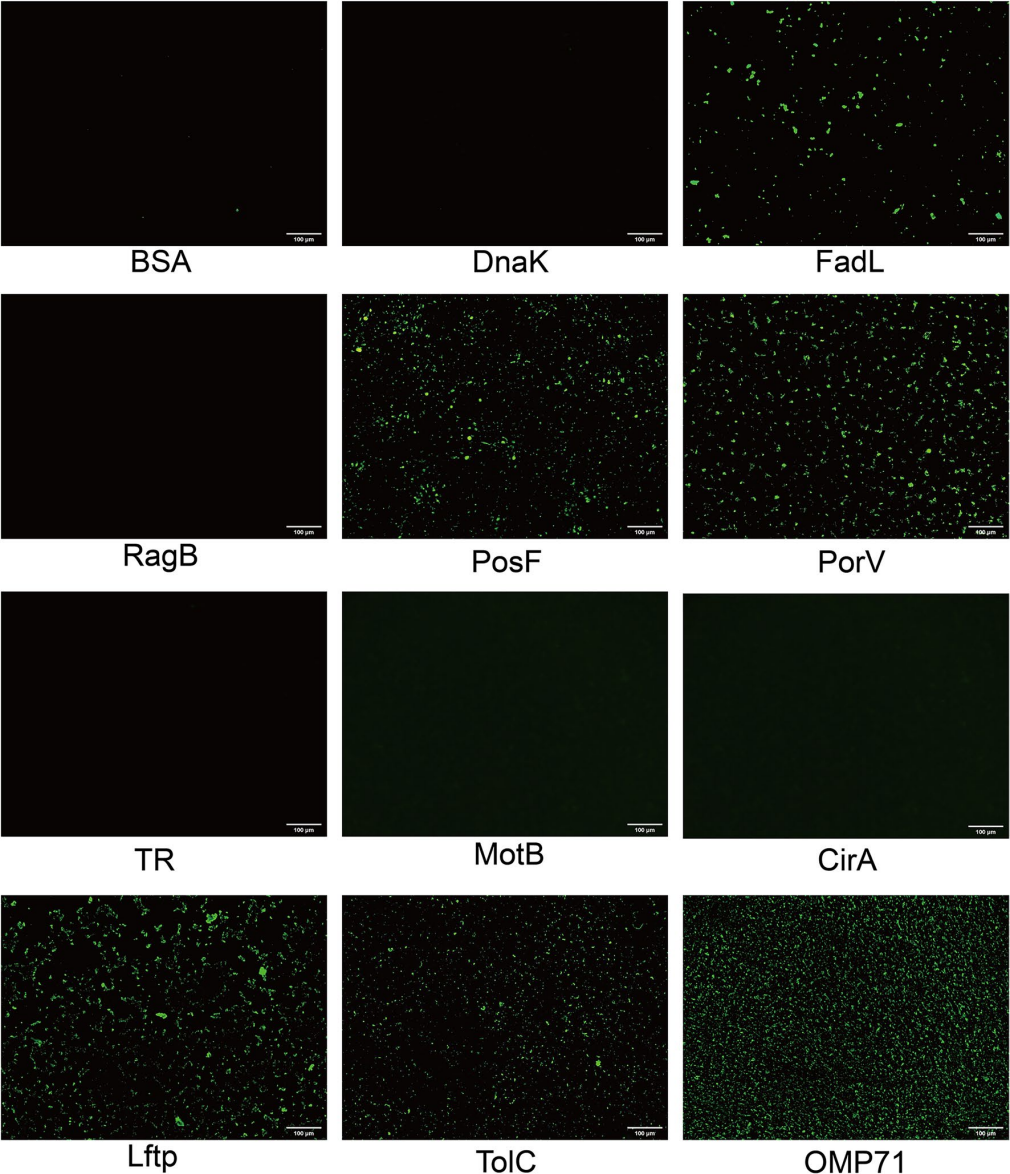
**Immunofluorescence detection of the adhesion of purified OMPs to DEF cells.**

OMPs were purified from *R. anatipestifer* RA-YM at a concentration of 5.4 mg/mL and detected by SDS‒PAGE (Figure 2A). These OMPs were pulled down from surface proteins of DEF cells that were either biotinylated (experimental group) or unbiotinylated (control group), and avidin agarose alone (negative control) was detected via SDS‒PAGE. More protein species were observed in the experimental group than in both the control and negative control groups (Figure 2B). The interacting OMPs from the preceding assays were subjected to mass spectrometry sequencing, which revealed ten bacterial proteins that bound to DEF cell surface proteins with high confidence: TR (KYF39_03505), TolC (KYF39_05115), MotB (KYF39_06840), DnaK (KYF39_02250), FadL (KYF39_00420), RagB (KYF39_07950), PorV (KYF39_00455), PosF (KYF39_03195), Lftp (KYF39_06330), and CirA (KYF39_03925) (Table 1). The ten proteins whose signal peptides were deleted were successfully expressed, purified and detected by SDS‒PAGE (Figure 3). The purified FadL, PosF, PorV, Lftp, and TolC proteins, along with OMP71 as described previously (41), adhered to DEF cells in indirect immunofluorescence assays (Figure 4). Therefore, antibodies against these proteins were prepared and examined by western blotting, which demonstrated the specificity of the antibodies when tested with total OMPs of *R. anatipestifer* RA-YM (Additional file 2).

**Table 1:** **Outer membrane proteins most likely act as adhesins**

Protein	GenBank	Sum PEP Score	Percent Cover	Peptides	Unique Peptides	MW (kD)
CirA	QXT33890.1	262.58	69	55	55	106.9
DnaK	QXT33579.1	3.56	12	3	2	67.8
FadL	QXT33230.1	14.62	15	7	7	47.2
RagB	QXT32740.1	3.66	11	5	5	49.4
Lftp	QXT32447	15.41	19	7	7	44.9
MotB	QXT32541	14.33	25	6	6	30.4
PorV	QXT33237.1	8.92	11	4	4	40.7
PosF	QXT33746.1	6.59	17	3	3	22.8
TolC	QXT32222.1	5.98	7	3	3	50.8
TR	QXT33806.1	8.14	15	5	5	35.8
OMP71	QXT33060.1	80.02	42	24	24	70.9

### PorV and PosF mediate the adhesion of *R. anatipestifer* to DEF cells

**Figure 5:**
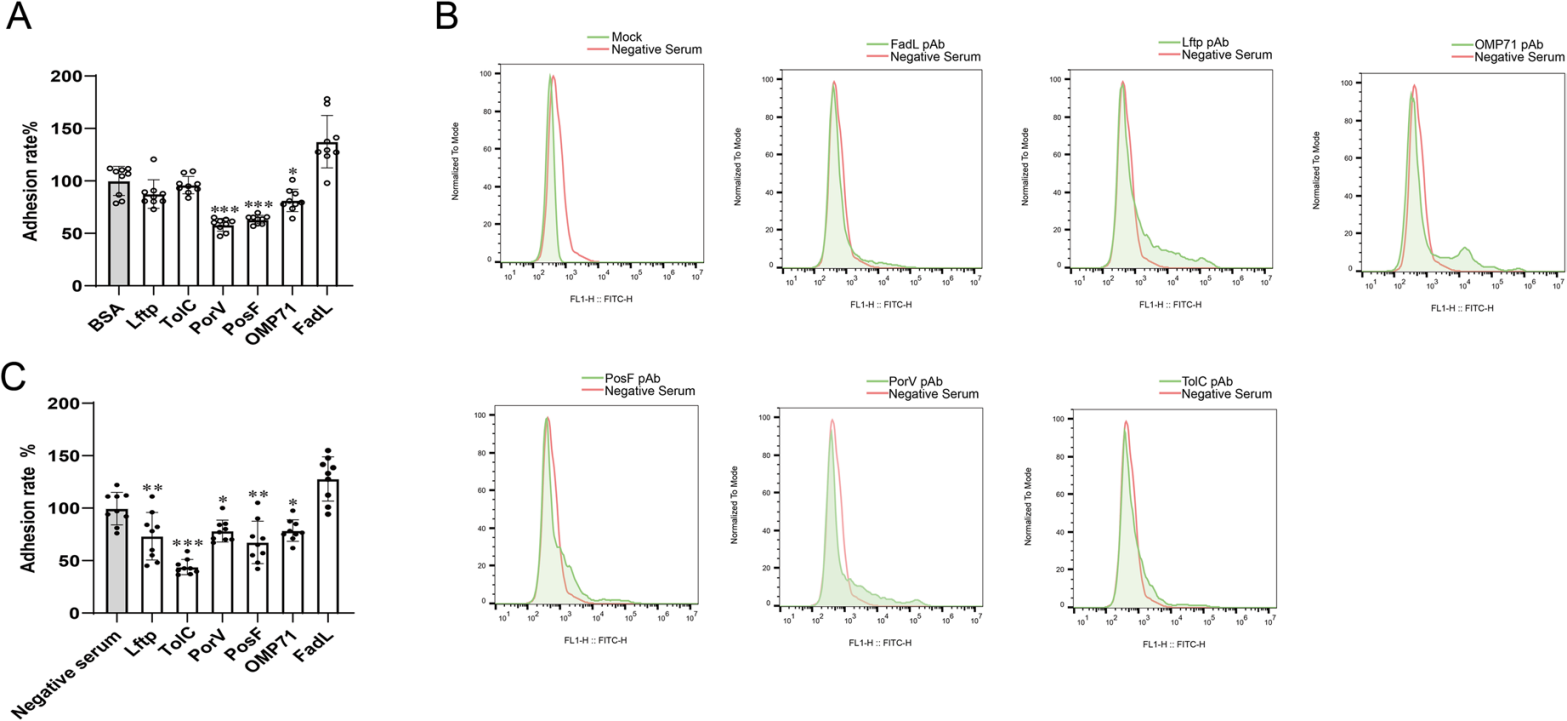
**Examination of *R. anatipestifer *RA-YM adhesins by competitive inhibition tests**. **A** Protein competition assays. **B** FACS detection of the surface location of adhesins in *R. anatipestifer* using a combination of OMPs and cognate pAbs. **C** Polyclonal antibody blocking assays. All the data are the mean values of three independent experiments that were analysed by one-way ANOVA. The error bars represent the standard deviations. *P ≤ 0.05; **P ≤ 0.01; ***P ≤ 0.001. The data points are biological repetitions.

The protein competition assays described above revealed that, among the six proteins analysed, PorV, PosF, and OMP71 significantly inhibited the adhesion of *R. anatipestifer* RA-YM to DEF cells (Figure 5A). The surface locations of the six proteins were verified by flow cytometry, which revealed that the extracellular domains of the proteins bound to the cognate antibodies (Figure 5B). Moreover, antibody blocking assays indicated that antibodies against PosF, PorV, Lftp, TolC, and OMP71 restrained the adhesion of *R. anatipestifer* to DEF cells (Figure 5C). Thus, PorV and PosF in particular appear to be novel surface adhesion factors of *R. anatipestifer* and were characterized further. The PorV and PosF proteins of strain RA-YM presented over 90% conservation, as determined using MEGA and GENEDOC, compared with homologous proteins from nine *R. anatipestifer* isolates available in the NCBI database (Additional file 3).

### Knockout of *porV* or *posF* reduces adhesion and invasion by *R. anatipestifer*

**Figure 6:**
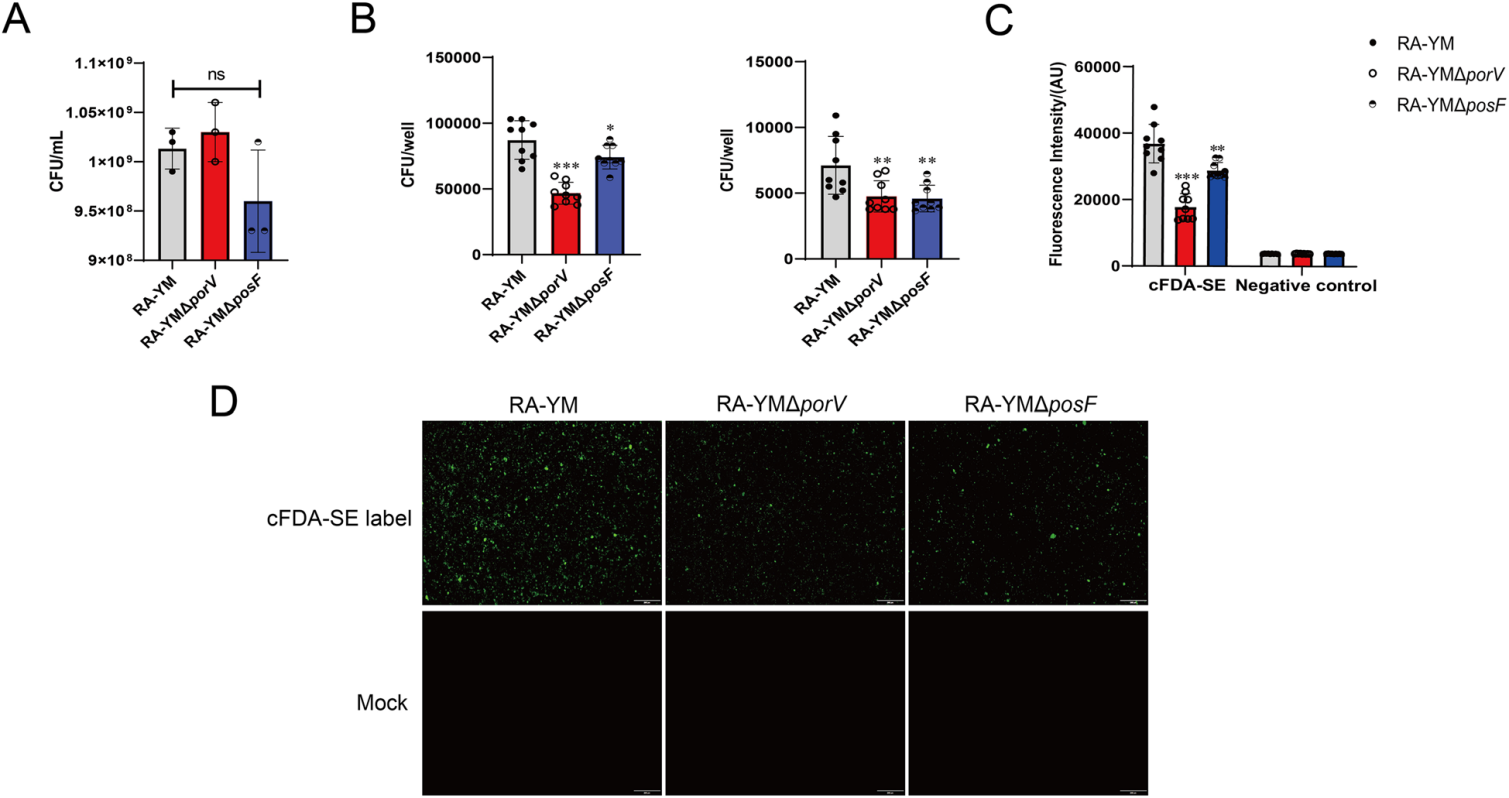
**Adhesion and invasion assays of *R. anatipestifer*** **RA-YM, RA-YM** **Δ*porV***, **and RA-YM Δ*posF*** **strains.** **A** Effects of the deletion of the *porV* and *posF* genes in the RA-YM strain on cell viability. **B** Left: Adhesion assays of RA-YM, RA-YM Δ*porV*, and RA-YM Δ*posF* with DEF cells. Right: Invasion assays of RA-YM, RA-YM Δ*porV*, and RA-YM Δ*posF* in DEF cells. **C** Adhesion of cFDA-SE-labelled *R. anatipestifer* RA-YM, RA-YM Δ*porV*, and RA-YM Δ*posF* to DEF cells was examined by fluorescence intensity. **D** Fluorescence microscopy images of the adhesion of cFDA-SE-labelled *R. anatipestifer* RA-YM, RA-YM Δ*porV*, and RA-YM Δ*posF* strains to DEF cells. Unlabelled *R. anatipestifer* RA-YM, RA-YM Δ*porV*, and RA-YM Δ*posF* were used as controls. All the data are the mean values of three independent experiments that were analysed by one-way ANOVA and two-way ANOVA. The error bars represent the standard deviations. *P ≤ 0.05; **P ≤ 0.01; ***P ≤ 0.001. The data points are biological repetitions.

Knockout of *porV* or *posF* was confirmed by PCR, which revealed that the genes were intact in the wild-type strain but absent in the mutant strains; these genes were replaced by the spectinomycin resistance marker gene (Additional file 4). The viabilities of the wild-type, Δ*porV*, and Δ*posF* strains on solid media were 1.05 × 10^9^/mL, 1.10 × 10^9^/mL, and 9.60 × 10^8^/mL, respectively, which were not significantly different and indicate that the genes are not essential under the test conditions (Figure 6A). However, the adhesion and invasion of DEF cells by *R. anatipestifer* were significantly reduced by *porV* or *posF* knockout (Figure 6B). This observation was verified by fluorescence analysis of binding to DEF cells (Figures 6C, D). Thus, PorV and PosF are vital cell adhesion factors in *R. anatipestifer*.

### Deletion of *porV* or *posF* attenuates the virulence of *R. anatipestifer*

**Figure 7:**
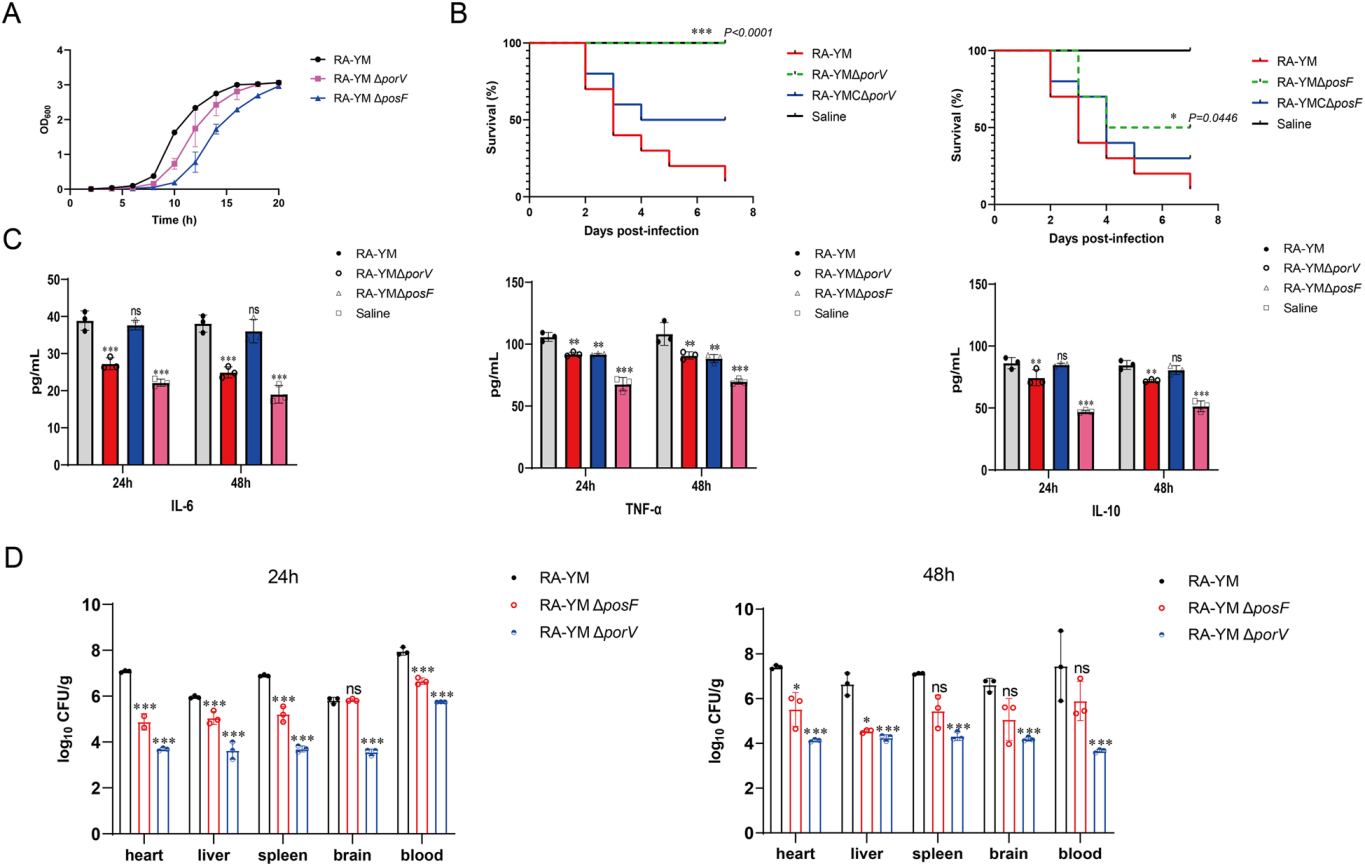
**Knockout of porV and posF reduces the pathogenicity of *R. anatipestifer*** **RA-YM in ducklings**. **A** Growth curves of *R. anatipestifer* RA-YM, RA-YM Δ*porV*, and RA-YM Δ*posF* strains in TSB. Standard deviations are depicted with coloured error bars. **B** Left: Survival rate of ducklings infected with the RA-YM, RA-YM Δ*porV*, and RA-YM CΔ*porV* strains. Right: Survival rates of ducklings infected with the RA-YM, RA-YM Δ*posF*, and RA-YM CΔ*posF* strains. Ten ten-day-old ducklings per group infected with 10^7^ CFU of bacteria were assessed for survival. Statistical significance was determined using a log-rank (Mantel‒Cox) test. **C** The levels of IL-6, TNF-α, and IL-10 in duck serum were determined by ELISA after 24 h and 48 h of infection. **D** Bacterial loads in duckling blood and tissues 24 h and 48 h after infection. Ten-day-old animals were infected with 10^7^ CFU of RA-YM, RA-YM Δ*porV,* or RA-YM Δ*posF*. Heart, liver, spleen, brain, and blood samples were collected at 24 h and 48 h after inoculation, and single colonies were counted after serial dilution and agar plating. Statistical significance was assessed using two-way ANOVA. The error bars represent standard deviations from three independent experiments. *P ≤ 0.05; **P ≤ 0.01; ***P ≤ 0.001.

The growth curves of the *R. anatipestifer* RA-YM, Δ*porV*, and Δ*posF* strains in TSB medium revealed that the deletion of *porV* or *posF* reduced the growth of *R. anatipestifer* (Figure 7A). The LD_50_ of *R. anatipestifer* RA-YM was 7.76 × 10^4^ CFU/mL, whereas the values for the Δ*porV* and Δ*posF* strains were 2.95 × 10^9^ CFU/mL and 1.00 × 10^6^ CFU/mL, respectively. In contrast, the LD_50_ values of the complemented strains RA-YM CΔ*porV* and RA-YM CΔ*posF* were 1.87 × 10^6^ CFU/mL and 3.71 × 10^5^ CFU/mL, respectively. Therefore, the deletion of the *porV* and *posF* genes decreased the virulence of *R. anatipestifer* by 3.8 × 10^4^-fold and 13-fold, respectively, but virulence was restored in both cases by supplying the deleted genes *in trans*. The survival rates of ducklings infected with the wild-type, deletion, and complemented strains (1.0 × 10^7^) were significantly lower (*P* < 0.05) than those of the wild-type strain (Figure 7B). These results demonstrate that PorV and PosF play important roles in *R. anatipestifer* infection.

### Deletion of the *porV *or* posF* genes weakens the production of inflammatory factors in ducklings

The impact of infection with *R. anatipestifer* on blood-related inflammatory factors in the duckling in vivo environment was assessed to further explore the infection process. Infection with the wild-type strain led to increases in the levels of the inflammatory cytokines IL-6, TNF-α, and IL-10, but deletion of the *porV* locus significantly reduced the levels of these factors (*P* ≤ 0.01) (Figure 7C). In contrast, the deletion of *posF* was accompanied by a decrease in TNF-α levels (*P* ≤ 0.01) but not in IL-6 or IL-10 levels. Thus, infection with a strain lacking *posF* still triggers an inflammatory reaction in infected animals, and as shown in the preceding experiments, *posF* deletion influences virulence less than deletion of the *porV* gene does.

### Knockout of *porV* or *posF* in *R. anatipestifer* reduces tissue bacterial loads and mitigates pathological lesions

**Figure 8:**
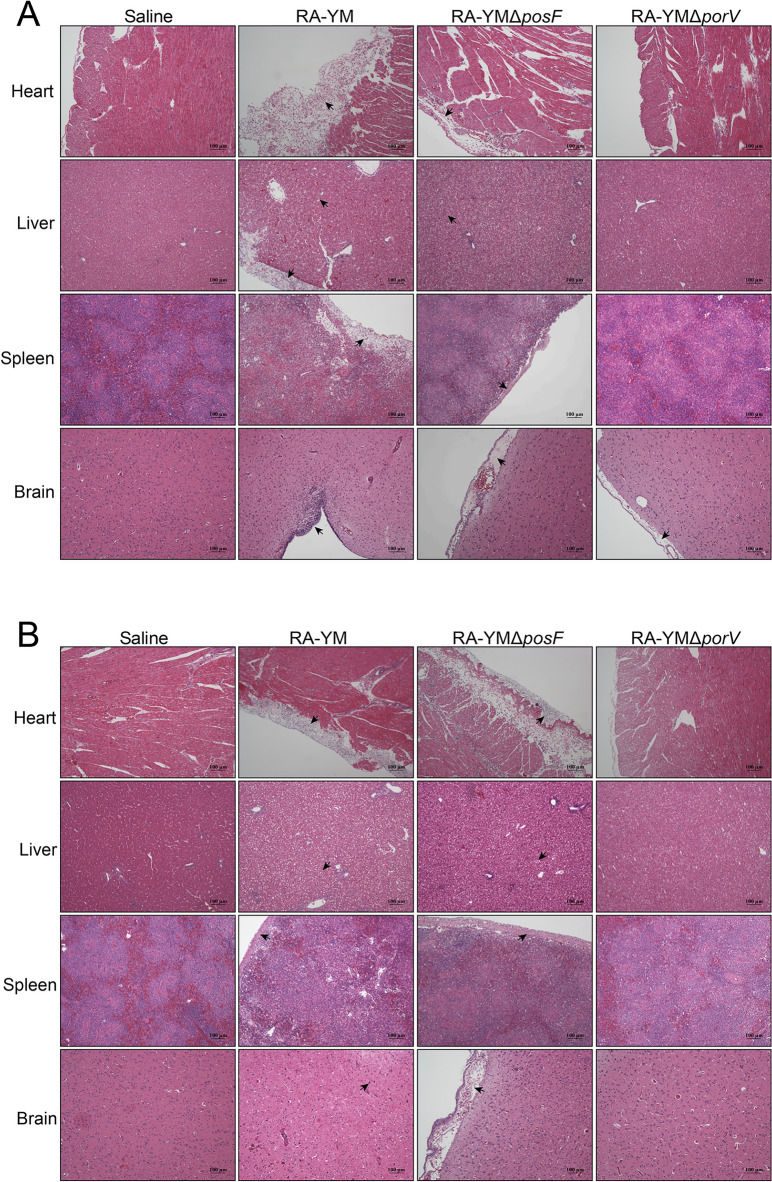
**Histopathological analysis of ducklings injected with saline, *R. anatipestifer*** **RA-YM, RA-YM Δ*posF*, or RA-YM Δ*porV*** **for 24 h (A) or 48 h (B).** The direction of the arrows indicates inflammation, including fatty degeneration, tissue serosa thickening, and inflammatory cell infiltration. Compared with the wild-type strain, the deletion of *porV* reduced the inflammatory response, whereas the deletion of *posF* had a weaker effect.

The preceding experiments revealed that *porV* and *posF* are virulence genes in *R. anatipestifer* RA-YM, which was further assessed by examining bacterial loads in the heart, liver, spleen, brain, and blood of ducklings at 24 h and 48 h after infection. The loads of the RA-YM Δ*porV* strain in these tissues were lower than those of the wild-type strain after both 24 h (*P* ≤ 0.01) and 48 h (*P* ≤ 0.01) of infection. The loads of the RA-YM Δ*posF* strain in the heart and liver were also lower than those of the wild-type strain at 24 h (*P* ≤ 0.01) and 48 h (*P* ≤ 0.05) (Figure 7D). Histopathological sections of the heart, liver, spleen, and brain of ducklings showed varying degrees of serous membrane thickening and inflammatory cell infiltration after challenge with RA-YM, and the liver showed typical fatty degeneration. Tissue damage was greatly reduced after the *porV* gene was knocked out, whereas *posF* deletion did not cause pronounced histopathological changes (Figure 8).

## Discussion

Large-scale protein interactome techniques have been developed that permit intensive probing of protein networks [42], including yeast two-hybrid analysis, tandem affinity purification, protein–fragment complementation assays, luminescence-based mammalian interactome analysis, mammalian protein–protein interaction traps, and affinity chromatography-based surface proteomics [43–48].

Yeast two-hybrid analysis allows simultaneous screening of many protein‒protein interactions and directly detects these interactions through reporter gene expression. However, this technique can produce false positives or negatives, may not accurately reflect the cellular environment, and may be unsuitable for membrane proteins. Tandem affinity purification enhances the enrichment of specific proteins and their interaction partners while reducing background noise. However, proteins need to be expressed and tagged, and the tags may interfere with protein function. Protein–fragment complementation assays enable the monitoring of protein–protein interactions in living cells, which better reflect the biological state. However, certain interactions may not effectively reconstitute the reporter or produce a sufficiently strong signal, potentially leading to false negatives. Luminescence-based mammalian interactome analysis is highly sensitive to fluorescence or luminescent signals and allows the detection of low-abundance protein interactions. However, this approach is applicable mainly to luminescent reporter genes and specific protein detection methods, making it unsuitable for all types of interactions. Mammalian protein–protein interaction traps are particularly suited for detecting protein interactions in mammalian cells and provide a system that closely mimics physiological conditions. However, this technique is highly dependent on protein expression and precise protein tagging, which renders it infeasible for certain proteins that are difficult to express or construct. Affinity chromatography-based surface proteomics enables the analysis of membrane protein interactions and is applicable to proteins on the cell surface and their interaction partners, thereby covering a broad range of proteins [48–53]. Using this method, surface proteins can be extracted simultaneously from both bacteria and host cells, which is highly convenient and, when combined with mass spectrometry, enables efficient analysis and identification of protein complexes.

**Figure 9:**
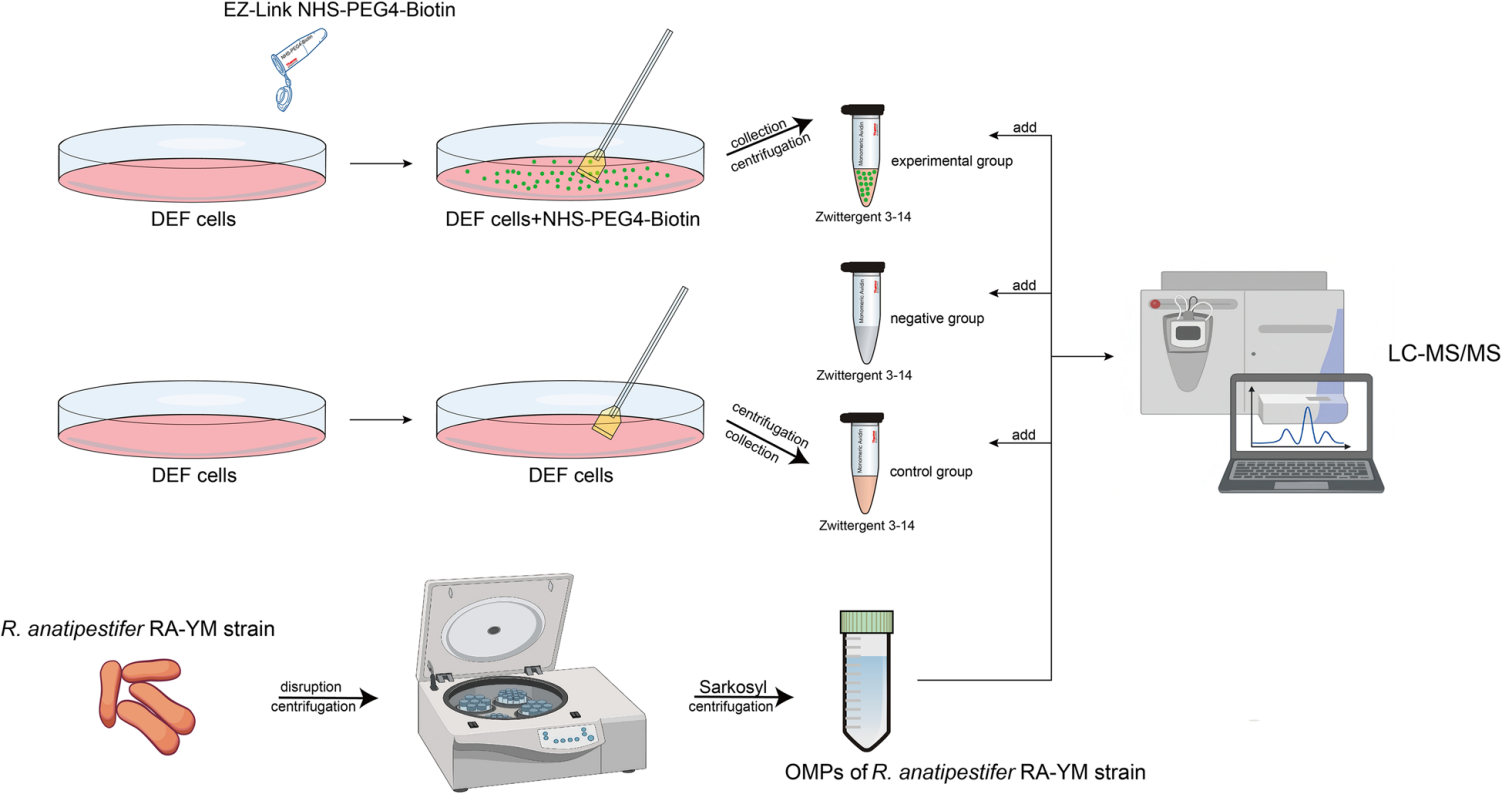
**Verification of biotinylation of surface molecules of DEF cells.**

Therefore, affinity chromatography-based surface proteomics was selected here for identifying surface adhesins of *R. anatipestifer*, although the standard NeutrAvidin Agarose resin was altered to Monomeric Avidin Agarose (Figure 9). Although the binding affinity of biotin for the latter resin is weakened, denaturation during elution is avoided, and the adverse effects on biotinylated proteins caused by the separation of avidin from the resin and coelution with the desired purified products are weakened. The candidate proteins identified by this approach were examined by mass spectrometry using peptide spectrum matches > 99%, unique peptides ≥ 1, and a protein fault tolerance rate > 1%, along with peptide coverage using relevant annotations from the GO, COG, IPR, and InterProScan databases (Figures 2C–E). This strategy generated ten candidate proteins (TR, TolC, MotB, DnaK, FadL, RagB, PorV, PosF, Lftp, and CirA) that were expressed and purified with His_10_-tags (Figure 3). The OmpA protein was previously implicated in adhesion and invasion by *R. anatipestifer* [11, 31], but this protein was not selected for in the present study. As there was no difference in the number of peptides obtained during the database searches, the OmpA protein was excluded from further analysis.

Direct adhesion assays of the candidate proteins with DEF cells excluded DnaK, RagB, TR, MotB, and CirA as potential adhesins. The OMP71 protein was employed as a positive control, as this factor interacted with the duck CD46 inhibitory complement receptor in our previous study [41]. Competitive inhibition assays of the remaining candidate proteins with polyclonal antibodies revealed that PorV and PosF are surface adhesins of *R. anatipestifer* RA-YM.

PorV, also known as LptO, is the most abundant component of the T9SS and was reported as a receptor of the sortase PorU. PorV is also required for protein secretion and plays a critical role in the covalent interaction with anionic lipopolysaccharides within the substrate of the T9SS [35, 54–57]. A PorV-deficient mutant of *Flavobacterium johnsoniae* was partially deficient in attachment due to the absence of certain other adhesins secreted by the T9SS [58]. In this study, we present the first evidence that PorV is involved in direct adhesion between *R. anatipestifer* and host cells. We also demonstrated that the PorV polyclonal antibody recognized the surface of *R. anatipestifer* (Figure 5B), which suggests that PorV is exposed on the OM, which provides the basis for the adhesin behavior of the protein. Therefore, identification of the receptor for PorV on the surface of DEF cells is crucial. According to existing studies, PorV interacts with the CTD of T9SS effector proteins [59]. We hypothesize that the receptor on DEF cells may also possess a CTD-like domain and play a key role in mediating the adhesion and invasion of *R. anatipestifer* into host cells.

PosF belongs to the porin superfamily of barrel-shaped transmembrane proteins and comprises eight reverse parallel β-folds with four extracellular loops, as predicted by the conserved domain database and the AlphaFold protein structure database. Porins of Gram-negative bacteria play diverse biological functions during bacterial‒host interactions and contribute to virulence in different pathogens [60–63]. Porins, including Aha of *Aeromonas veronii*, porin P2 of *Haemophilus influenzae*, and OmpF and OmpC of uropathogenic *E. coli*, also participate in adhesion [64–66], which further supports the role of PosF as an adhesin in *R. anatipestifer*.

The Lftp, TolC, and FadL proteins were not identified as primary surface adhesins of *R. anatipestifer;* although the antibodies against Lftp and TolC blocked the binding of the bacterium to DEF cells, the recombinant Lftp and TolC proteins did not impair adhesion (*P* > 0.05). Neither the antibody nor the FadL protein inhibited the adhesion of *R. anatipestifer* to DEF cells (*P* > 0.05). FadL binds to DEF cells at its intracellular sites so that only the recombinant FadL protein can adhere to DEF cells. In the case of Lftp, the majority of the protein may be buried by lipopolysaccharides in *R. anatipestifer* and therefore may be difficult to access. Although the TolC protein has been demonstrated to be an adhesin in *Vibrio harveyi* [67], the recombinant TolC protein blocked adhesion only weakly in *R. anatipestifer*, with no significant difference (*P* > 0.05). Moreover, the lower expression of TolC on the surface of *R. anatipestifer* may explain the inability of this protein to act as an inhibitor (Figure 5B).

As is the case with most pathogenic microbes, infection of ducklings with *R. anatipestifer* leads to a surge in cytokine levels and inflammation, which reflects disruption of the balance between proinflammatory and anti-inflammatory cytokines in the host [68, 69]. The levels of inflammatory factors decreased significantly when the *porV* gene was knocked out in *R. anatipestifer* (Figure 7C). In contrast, the deletion of *posF* had a weaker effect on both cytokine levels and tissue damage (Figure 7C and Figure 8). Although PosF may be less critical for virulence than PorV is, the protein nevertheless may play a minor role in the pathogenicity of *R. anatipestifer*. However, the deletion of *porV* also led to a decrease in T9SS-related protein secretion, which may also influence virulence in *R. anatipestifer*.

Most adhesins of Gram-negative bacteria, such as adhesin A (NadA) of *Neisseria meningitidis*, Hia of *H. influenzae*, and SadA of *Salmonella* [70–72], are anchored in the OM and have exposed sites on the surface, which results in excellent immunogenicity [73]. The discovery here of highly conserved surface adhesins of *R. anatipestifer* suggests that PorV and PosF may form the basis of new subunit vaccines to replace current inactivated and attenuated vaccines and simultaneously solve the weakness of cross-protection among serotypes provided by existing therapies.

## Supplementary Information

**Additional file 1: The bacterial strains, plasmids and primers used in this study.**

**Additional file 2: The specificity of each antibody was detected by western blotting.** Substrate: Total OMPs of the *R. anatipestifer* RA-YM strain. The following antibodies were used: rabbit polyclonal antibodies against TolC, PorV, Lftp, PosF, FadL, and OMP71.

**Additional file 3: Conservation analysis of PorV and PosF proteins in different** ***R. anatipestifer*** isolates. Conservation of PorV (A) and PosF (B) in different *R. anatipestifer* isolates analysed by MEGA and GENEDOC.

**Additional file 4: Construction of RA-YMΔ*porV*, RA-YMCΔ*porV*, RA-YMΔ*posF***, **and RA-YMCΔ*posF***. (A) 1. Amplification of the *posF* gene (624 bp) in RA-YM. 2. Amplification of the *posF* gene in RA-YMΔ*posF*. 3. Amplification of the *spc* gene without a promoter (726 bp) in RA-YM. 4. Amplification of the *spc* gene without a promoter in RA-YM. M: DL2000 DNA Marker. 5. Amplification of the *porV* gene (1137 bp) in RA-YM. 6. Amplification of the *porV* gene in RA-YMΔ*porV*. 7. Amplification of the *spc* gene without a promoter in RA-YM. 8. Amplification of the *spc* gene without a promoter in RA-YMΔ*porV*. (B) 1. Amplification of the promoter of the *porV* gene (335 bp). 2. Amplification of the *porV* gene (1137 bp). 3. Amplification of the promoter of the *posF* gene (344 bp). 4. Amplification of the *posF* gene (624 bp). M: DL2000 DNA Marker.

## Data Availability

The original contributions presented in the study are included in the article and Supplementary Materials. Further inquiries can be directed to the corresponding author. The raw mass spectrometry data have been deposited in ProteomeXchange via the iProX partner repository with the accession number PXD057236.

## References

[1] Leavitt S, Ayroud M (1997) *Riemerella anatipestifer* infection of domestic ducklings. Can Vet J 38:113 9028598 PMC1576529

[2] Cheng A, Wang M, Chen X, Zhu D, Huang C, Liu F, Zhou Y, Guo Y, Liu Z, Fang P (2003) Epidemiology and new serotypes of *Riemerella anatipestifer* isolated from ducks in China and studies on their pathogenic characteristics. Chin J Vet Sci 78:1469–1473

[3] Hu Q, Zhang Z, Miao J, Liu Y, Liu X, Ding S (2001) The epidemiology study of *Riemerella anatipestifer* infection in Jiangsu and Anhui provinces. Chin Vet Sci 31:12–13

[4] Segers P, Mannheim W, Vancanneyt M, De Brandt K, Hinz KH, Kersters K, Vandamme P (1993) *Riemerella anatipestifer* gen. nov., comb. nov., the causative agent of septicemia anserum exsudativa, and its phylogenetic affiliation within the Flavobacterium-Cytophaga rRNA homology group. Int J Syst Bacteriol 43:768–776 8240957 10.1099/00207713-43-4-768

[5] Yehia N, Salem HM, Mahmmod Y, Said D, Samir M, Mawgod SA, Sorour HK, AbdelRahman MAA, Selim S, Saad AM, El-Saadony MT, El-Meihy RM, Abd El-Hack ME, El-Tarabily KA, Zanaty AM (2023) Common viral and bacterial avian respiratory infections: an updated review. Poult Sci 102:102553 36965253 10.1016/j.psj.2023.102553 PMC10064437

[6] Wang J, Ma Q, Wang S, Liu D, Xu M, Zhao J, Zhang Z, Zou Z, Liu Z, Wei S, Li Y (2024) Isolation, identification and pathogenicity study of a strain of *Riemerella anatipestifer* originating from broiler breeders. Chin Poult 46:107–112

[7] Li X, Zhao W, Song J, Zhu J, Yang S (2021) Isolation and identification of *Riemerella anatipestifer* from chickens and genetic evolution analysis of *ompA* gene. Chin J Vet Sci 41:253–258

[8] Pathanasophon P, Sawada T, Pramoolsinsap T, Tanticharoenyos T (1996) Immunogenicity of *Riemerella anatipestifer* broth culture bacterin and cell-free culture filtrate in ducks. Avian Pathol 25:705–719 18645893 10.1080/03079459608419176

[9] Timms LM, Marshall RN (1989) Laboratory assessment of protection given by experimental *Pasteurella anatipestifer* vaccine. Br Vet J 145:483–493 2790442 10.1016/0007-1935(89)90059-6

[10] Subramaniam S, Huang B, Loh H, Kwang J, Tan HM, Chua KL, Frey J (2000) Characterization of a predominant immunogenic outer membrane protein of *Riemerella anatipestifer*. Clin Diagn Lab Immunol 7:168–174 10702488 10.1128/cdli.7.2.168-174.2000 PMC95844

[11] Hu Q, Han X, Zhou X, Ding C, Zhu Y, Yu S (2011) OmpA is a virulence factor of *Riemerella anatipestifer*. Vet Microbiol 150:278–283 21349662 10.1016/j.vetmic.2011.01.022

[12] Dou Y, Wang X, Yu G, Wang S, Tian M, Qi J, Li T, Ding C, Yu S (2017) Disruption of the M949_RS01915 gene changed the bacterial lipopolysaccharide pattern, pathogenicity and gene expression of *Riemerella anatipestifer*. Vet Res 48:6 28166822 10.1186/s13567-017-0409-6 PMC5294843

[13] Yi H, Yuan B, Liu J, Zhu D, Wu Y, Wang M, Jia R, Sun K, Yang Q, Chen S, Liu M, Chen X, Cheng A (2017) Identification of a wza-like gene involved in capsule biosynthesis, pathogenicity and biofilm formation in *Riemerella anatipestifer*. Microb Pathog 107:442–450 28442426 10.1016/j.micpath.2017.04.023

[14] Zhang Y, Wang Y, Zhang Y, Jia X, Li C, Zhou Z, Hu S, Li Z (2022) Genome-wide analysis reveals that PhoP regulates pathogenicity in *Riemerella anatipestifer*. Microbiol Spectr 10:e0188322 36197298 10.1128/spectrum.01883-22 PMC9603813

[15] Guo Y, Hu D, Guo J, Wang T, Xiao Y, Wang X, Li S, Liu M, Li Z, Bi D, Zhou Z (2017) *Riemerella anatipestifer* Type IX secretion system is required for virulence and gelatinase secretion. Front Microbiol 8:2553 29312236 10.3389/fmicb.2017.02553 PMC5742166

[16] Sandoz KM, Moore RA, Beare PA, Patel AV, Smith RE, Bern M, Hwang H, Cooper CJ, Priola SA, Parks JM, Gumbart JC, Mesnage S, Heinzen RA (2021) β-Barrel proteins tether the outer membrane in many Gram-negative bacteria. Nat Microbiol 6:19–26 33139883 10.1038/s41564-020-00798-4 PMC7755725

[17] Hogenkamp A, Herías MV, Tooten PC, Veldhuizen EJ, Haagsman HP (2007) Effects of surfactant protein D on growth, adhesion and epithelial invasion of intestinal Gram-negative bacteria. Mol Immunol 44:3517–3527 17477970 10.1016/j.molimm.2007.03.013

[18] Confer AW, Ayalew S (2013) The OmpA family of proteins: roles in bacterial pathogenesis and immunity. Vet Microbiol 163:207–222 22986056 10.1016/j.vetmic.2012.08.019

[19] Krishnan S, Prasadarao NV (2012) Outer membrane protein A and OprF: versatile roles in Gram-negative bacterial infections. FEBS J 279:919–931 22240162 10.1111/j.1742-4658.2012.08482.x PMC3338869

[20] He X, Jiang K, Xiao J, Lian S, Chen Y, Wu R, Wang L, Sun D, Guo D (2022) Interaction of 43K OMP of *Fusobacterium necrophorum* with fibronectin mediates adhesion to bovine epithelial cells. Vet Microbiol 266:109335 35121302 10.1016/j.vetmic.2022.109335

[21] Sebbane F, Uversky VN, Anisimov AP (2020) *Yersinia pestis* plasminogen activator. Biomolecules 10:1554 33202679 10.3390/biom10111554 PMC7696990

[22] Kaur D, Mukhopadhaya A (2020) Outer membrane protein OmpV mediates *Salmonella* enterica serovar typhimurium adhesion to intestinal epithelial cells via fibronectin and α1β1 integrin. Cell Microbiol 22:e13172 32017350 10.1111/cmi.13172

[23] Smani Y, Dominguez-Herrera J, Pachón J (2013) Association of the outer membrane protein Omp33 with fitness and virulence of *Acinetobacter baumannii*. J Infect Dis 208:1561–1570 23908480 10.1093/infdis/jit386

[24] Sadarangani M, Pollard AJ, Gray-Owen SD (2011) Opa proteins and CEACAMs: pathways of immune engagement for pathogenic *Neisseria*. FEMS Microbiol Rev 35:498–514 21204865 10.1111/j.1574-6976.2010.00260.x

[25] Ryll M, Christensen H, Bisgaard M, Christensen JP, Hinz KH, Köhler B (2001) Studies on the prevalence of *Riemerella anatipestifer* in the upper respiratory tract of clinically healthy ducklings and characterization of untypable strains. J Vet Med B Infect Dis Vet Public Health 48:537–546 11666036 10.1046/j.1439-0450.2001.00471.x

[26] Li S, Gong X, Chen Q, Zheng F, Ji G, Liu Y (2018) Threshold level of *Riemerella anatipestifer* crossing blood-brain barrier and expression profiles of immune-related proteins in blood and brain tissue from infected ducks. Vet Immunol Immunopathol 200:26–31 29776609 10.1016/j.vetimm.2018.04.005

[27] Huang G, Yang S, Long T, Gao Y, Lin G (2024) Proteomic analysis of brain tissue from ducks with meningitis caused by *Riemerella anatipestifer* infection. Poult Sci 103:104059 39068696 10.1016/j.psj.2024.104059 PMC11338091

[28] Chen Z, Zhu M, Liu D, Wu M, Niu P, Yu Y, Ding C, Yu S (2024) Occludin and collagen IV degradation mediated by the T9SS effector SspA contributes to blood-brain barrier damage in ducks during *Riemerella anatipestifer* infection. Vet Res 55:49 38594770 10.1186/s13567-024-01304-y PMC11005161

[29] Gao Q, Lu S, Wang M, Jia R, Chen S, Zhu D, Liu M, Zhao X, Yang Q, Wu Y, Zhang S, Huang J, Mao S, Ou X, Sun D, Tian B, Cheng A (2021) Putative *Riemerella anatipestifer* outer membrane protein H affects virulence. Front Microbiol 12:708225 34616377 10.3389/fmicb.2021.708225 PMC8488386

[30] Li S, Wang Y, Yang R, Zhu X, Bai H, Deng X, Bai J, Zhang Y, Xiao Y, Li Z, Liu Z, Zhou Z (2023) Outer membrane protein OMP76 of *Riemerella anatipestifer* contributes to complement evasion and virulence by binding to duck complement factor vitronectin. Virulence 14:2223060 37326479 10.1080/21505594.2023.2223060 PMC10281475

[31] Zou R, Wu X, Chen Q, Gong X, Chu Y, Zheng F (2023) OmpA is involved in the invasion of duck brain microvascular endothelial cells by *Riemerella anatipestifer*. Vet Microbiol 280:109692 36863175 10.1016/j.vetmic.2023.109692

[32] Chen Z, Wang X, Ren X, Han W, Malhi KK, Ding C, Yu S (2019) *Riemerella anatipestifer* GldM is required for bacterial gliding motility, protein secretion, and virulence. Vet Res 50:43 31164171 10.1186/s13567-019-0660-0 PMC6549377

[33] Sato K, Naito M, Yukitake H, Hirakawa H, Shoji M, McBride MJ, Rhodes RG, Nakayama K (2010) A protein secretion system linked to bacteroidete gliding motility and pathogenesis. Proc Natl Acad Sci 107:276–281 19966289 10.1073/pnas.0912010107 PMC2806738

[34] McBride MJ, Zhu Y (2013) Gliding motility and Por secretion system genes are widespread among members of the phylum bacteroidetes. J Bacteriol 195:270–278 23123910 10.1128/JB.01962-12 PMC3553832

[35] Sato K, Yukitake H, Narita Y, Shoji M, Naito M, Nakayama K (2013) Identification of *Porphyromonas gingivalis* proteins secreted by the Por secretion system. FEMS Microbiol Lett 338:68–76 23075153 10.1111/1574-6968.12028

[36] Shrivastava A, Johnston JJ, van Baaren JM, McBride MJ (2013) *Flavobacterium johnsoniae* GldK, GldL, GldM, and SprA are required for secretion of the cell surface gliding motility adhesins SprB and RemA. J Bacteriol 195:3201–3212 23667240 10.1128/JB.00333-13 PMC3697645

[37] Yang R, Li S, Guo J, Wang Y, Dong Z, Wang Q, Bai H, Ning C, Zhu X, Bai J, Hu S, Xiao Y, Li Z, Zhou Z (2024) Serine protease RAYM_01812 (SspA) inhibits complement-mediated killing and monocyte chemotaxis and contributes to virulence of *riemerella anatipestifer* in ducks. Virulence 25:2421219 10.1080/21505594.2024.2421219 PMC11540087 39450484

[38] Hu D, Guo Y, Guo J, Wang Y, Pan Z, Xiao Y, Wang X, Hu S, Liu M, Li Z, Bi D, Zhou Z (2019) Deletion of the *Riemerella anatipestifer* type IX secretion system gene sprA results in differential expression of outer membrane proteins and virulence. Avian Pathol 48:191–203 30640518 10.1080/03079457.2019.1566594

[39] Guo Y, Hu D, Guo J, Li X, Guo J, Wang X, Xiao Y, Jin H, Liu M, Li Z, Bi D, Zhou Z (2017) The role of the regulator Fur in gene regulation and virulence of *Riemerella anatipestifer* assessed using an unmarked gene deletion system. Front Cell Infect Microbiol 7:382 28971067 10.3389/fcimb.2017.00382 PMC5609570

[40] Li L, Zhu DK, Zhou Y, Wang MS, Cheng AC, Jia RY, Chen S, Liu F, Yang QM, Chen XY (2012) Adhesion and invasion to duck embryo fibroblast cells by *Riemerella anatipestifer*. Poult Sci 91:3202–3208 23155031 10.3382/ps.2012-02552

[41] Wang Y, Li S, Ning C, Yang R, Wu Y, Cheng X, Xu J, Wang Y, Liu F, Zhang Y, Hu S, Xiao Y, Li Z, Zhou Z (2024) The outer membrane protein, OMP71, of *Riemerella anatipestifer*, mediates adhesion and virulence by binding to CD46 in ducks. Vet Res 55:138 39407352 10.1186/s13567-024-01393-9 PMC11481396

[42] Lievens S, Eyckerman S, Lemmens I, Tavernier J (2010) Large-scale protein interactome mapping: strategies and opportunities. Expert Rev Proteomics 7:679–690 20973641 10.1586/epr.10.30

[43] Fields S, Song O (1989) A novel genetic system to detect protein-protein interactions. Nature 340:245–246 2547163 10.1038/340245a0

[44] Ewing RM, Chu P, Elisma F, Li H, Taylor P, Climie S, McBroom-Cerajewski L, Robinson MD, O’Connor L, Li M, Taylor R, Dharsee M, Ho Y, Heilbut A, Moore L, Zhang S, Ornatsky O, Bukhman YV, Ethier M, Sheng Y, Vasilescu J, Abu-Farha M, Lambert JP, Duewel HS, Stewart II, Kuehl B, Hogue K, Colwill K, Gladwish K, Muskat B, Kinach R, Adams SL, Moran MF, Morin GB, Topaloglou T, Figeys D (2007) Large-scale mapping of human protein-protein interactions by mass spectrometry. Mol Syst Biol 3:89 17353931 10.1038/msb4100134 PMC1847948

[45] Tarassov K, Messier V, Landry CR, Radinovic S, Serna Molina MM, Shames I, Malitskaya Y, Vogel J, Bussey H, Michnick SW (2008) An in vivo map of the yeast protein interactome. Science 320:1465–1470 18467557 10.1126/science.1153878

[46] Braun P, Tasan M, Dreze M, Barrios-Rodiles M, Lemmens I, Yu H, Sahalie JM, Murray RR, Roncari L, de Smet AS, Venkatesan K, Rual JF, Vandenhaute J, Cusick ME, Pawson T, Hill DE, Tavernier J, Wrana JL, Roth FP, Vidal M (2009) An experimentally derived confidence score for binary protein-protein interactions. Nat Methods 6:91–97 19060903 10.1038/nmeth.1281 PMC2976677

[47] Lievens S, Vanderroost N, Van der Heyden J, Gesellchen V, Vidal M, Tavernier J (2009) Array MAPPIT: high-throughput interactome analysis in mammalian cells. J Proteome Res 8:877–886 19159283 10.1021/pr8005167

[48] Chen B, Zhang A, Xu Z, Li R, Chen H, Jin M (2011) Large-scale identification of bacteria-host crosstalk by affinity chromatography: capturing the interactions of *Streptococcus suis* proteins with host cells. J Proteome Res 10:5163–5174 21942651 10.1021/pr200758q

[49] Causier B, Davies B (2002) Analysing protein-protein interactions with the yeast two-hybrid system. Plant Mol Biol 50:855–870 12516858 10.1023/a:1021214007897

[50] Bauer A, Kuster B (2003) Affinity purification-mass spectrometry. Powerful tools for the characterization of protein complexes. Eur J Biochem 270:570–578 12581197 10.1046/j.1432-1033.2003.03428.x

[51] Blaszczak E, Lazarewicz N, Sudevan A, Wysocki R, Rabut G (2021) Protein-fragment complementation assays for large-scale analysis of protein-protein interactions. Biochem Soc Trans 49:1337–1348 34156434 10.1042/BST20201058 PMC8286835

[52] Barrios-Rodiles M, Brown KR, Ozdamar B, Bose R, Liu Z, Donovan RS, Shinjo F, Liu Y, Dembowy J, Taylor IW, Luga V, Przulj N, Robinson M, Suzuki H, Hayashizaki Y, Jurisica I, Wrana JL (2005) High-throughput mapping of a dynamic signaling network in mammalian cells. Science 307:1621–1625 15761153 10.1126/science.1105776

[53] Lemmens I, Lievens S, Tavernier J (2015) MAPPIT, a mammalian two-hybrid method for in-cell detection of protein-protein interactions. Methods Mol Biol 1278:447–455 25859968 10.1007/978-1-4939-2425-7_29

[54] Glew MD, Veith PD, Chen D, Gorasia DG, Peng B, Reynolds EC (2017) PorV is an outer membrane shuttle protein for the Type IX secretion system. Sci Rep 7:8790 28821836 10.1038/s41598-017-09412-w PMC5562754

[55] Gorasia DG, Glew MD, Veith PD, Reynolds EC (2020) Quantitative proteomic analysis of the type IX secretion system mutants in *Porphyromonas gingivalis*. Mol Oral Microbiol 35:78–84 32040252 10.1111/omi.12283

[56] Lauber F, Deme JC, Lea SM, Berks BC (2018) Type 9 secretion system structures reveal a new protein transPosF mechanism. Nature 564:77–82 30405243 10.1038/s41586-018-0693-y PMC6927815

[57] Chen YY, Peng B, Yang Q, Glew MD, Veith PD, Cross KJ, Goldie KN, Chen D, O’Brien-Simpson N, Dashper SG, Reynolds EC (2011) The outer membrane protein LptO is essential for the O-deacylation of LPS and the co-ordinated secretion and attachment of A-LPS and CTD proteins in *Porphyromonas gingivalis*. Mol Microbiol 79:1380–1401 21244528 10.1111/j.1365-2958.2010.07530.x

[58] Kharade SS, McBride MJ (2015) *Flavobacterium johnsoniae* PorV is required for secretion of a subset of proteins targeted to the type IX secretion system. J Bacteriol 197:147–158 25331433 10.1128/JB.02085-14 PMC4288674

[59] Dorgan B, Liu Y, Wang S, Aduse-Opoku J, Whittaker SB, Roberts MAJ, Lorenz CD, Curtis MA, Garnett JA (2022) Structural Model of a *Porphyromonas gingivalis* type IX Secretion System Shuttle Complex. J Mol Biol 434:167871 36404438 10.1016/j.jmb.2022.167871

[60] Di Donato A, Draetta GF, Illiano G, Tufano MA, Sommese L, Galdiero F (1986) Do porins inhibit the macrophage phagocyting activity by stimulating the adenylate cyclase? J Cyclic Nucleotide Protein Phosphor Res 11:87–97 2426319

[61] Galdiero M, Vitiello M, Galdiero S (2003) Eukaryotic cell signaling and transcriptional activation induced by bacterial porins. FEMS Microbiol Lett 226:57–64 13129608 10.1016/S0378-1097(03)00562-7

[62] Poole K (2002) Outer membranes and efflux: the path to multidrug resistance in Gram-negative bacteria. Curr Pharm Biotechnol 3:77–98 12022261 10.2174/1389201023378454

[63] Sharma A, Yadav SP, Sarma D, Mukhopadhaya A (2022) Modulation of host cellular responses by gram-negative bacterial porins. Adv Protein Chem Struct Biol 128:35–77 35034723 10.1016/bs.apcsb.2021.09.004

[64] Song HC, Kang YH, Zhang DX, Chen L, Qian AD, Shan XF, Li Y (2019) Great effect of porin(aha) in bacterial adhesion and virulence regulation in *Aeromonas veronii*. Microb Pathog 126:269–278 30399439 10.1016/j.micpath.2018.11.002

[65] Vitiello M, Finamore E, Cantisani M, Bevilacqua P, Incoronato N, Falanga A, Galdiero E, Galdiero M (2011) P2 porin and loop L7 from *Haemophilus influenzae* modulate expression of IL-6 and adhesion molecules in astrocytes. Microbiol Immunol 55:347–356 21288261 10.1111/j.1348-0421.2011.00318.x

[66] Beck CM, Willett JL, Cunningham DA, Kim JJ, Low DA, Hayes CS (2016) CdiA Effectors from Uropathogenic *Escherichia coli* Use Heterotrimeric Osmoporins as Receptors to Recognize Target Bacteria. PLoS Pathog 12:e1005925 27723824 10.1371/journal.ppat.1005925 PMC5056734

[67] Zhu Z, Dong C, Weng S, He J (2019) Identification of outer membrane protein TolC as the major adhesin and potential vaccine candidate for *Vibrio harveyi* in hybrid grouper, Epinephelus fuscoguttatus (♀) × E. lanceolatus (♂). Fish Shellfish Immunol 86:143–151 30453046 10.1016/j.fsi.2018.11.037

[68] Bahrami B, Macfarlane S, Macfarlane GT (2011) Induction of cytokine formation by human intestinal bacteria in gut epithelial cell lines. J Appl Microbiol 110:353–363 21070518 10.1111/j.1365-2672.2010.04889.x

[69] Gasparini R, Amicizia D, Lai PL, Panatto D (2012) *Neisseria meningitidis*, pathogenetic mechanisms to overcome the human immune defences. J Prev Med Hyg 53:50–55 23240160

[70] Liguori A, Dello Iacono L, Maruggi G, Benucci B, Merola M, Lo Surdo P, López-Sagaseta J, Pizza M, Malito E, BottomLey MJ (2018) NadA3 structures reveal undecad coiled coils and LOX1 binding regions competed by *Meningococcus* B vaccine-elicited human antibodies. mBio 9:e01914-18 30327444 10.1128/mBio.01914-18 PMC6191539

[71] Winter LE, Barenkamp SJ (2017) Immunogenicity of Nontypeable *Haemophilus influenzae* outer membrane vesicles and protective ability in the chinchilla model of otitis media. Clin Vaccine Immunol 24:e00138-e217 28768669 10.1128/CVI.00138-17 PMC5629667

[72] Raghunathan D, Wells TJ, Morris FC, Shaw RK, Bobat S, Peters SE, Paterson GK, Jensen KT, Leyton DL, Blair JM, Browning DF, Pravin J, Flores-Langarica A, Hitchcock JR, Moraes CT, Piazza RM, Maskell DJ, Webber MA, May RC, MacLennan CA, Piddock LJ, Cunningham AF, Henderson IR (2011) SadA, a trimeric autotransporter from *Salmonella enterica* serovar *Typhimurium*, can promote biofilm formation and provides limited protection against infection. Infect Immun 79:4342–4352 21859856 10.1128/IAI.05592-11 PMC3257908

[73] Thibau A, Dichter AA, Vaca DJ, Linke D, Goldman A, Kempf VAJ (2020) Immunogenicity of trimeric autotransporter adhesins and their potential as vaccine targets. Med Microbiol Immunol 209:243–263 31788746 10.1007/s00430-019-00649-y PMC7247748

