# Correction: PorV factor of the type IX secretion system and PosF porin act as adhesins in *Riemerella anatipestifer* infection

**DOI:** 10.1186/s13567-025-01584-y

**Published:** 2025-08-08

**Authors:** Sen Li, Yanhua Wang, Congran Ning, Rongkun Yang, Yaxin Wu, Xu Cheng, Keke Ren, Minghua Huang, Xiong Liu, Naiji Zhou, Wanpo Zhang, Sishun Hu, Yuncai Xiao, Zili Li, Hongbo Zhou, Zhengfei Liu, Zutao Zhou

**Affiliations:** 1 College of Veterinary Medicine, Huazhong Agricultural University, Wuhan, China; 2 State Key Laboratory of Agricultural Microbiology, Huazhong Agricultural University, Wuhan, China; 3 Key Laboratory of Preventive Veterinary Medicine in Hubei Province, Huazhong Agricultural University, Wuhan, China; 4 Hubei Hongshan Laboratory, Huazhong Agricultural University, Wuhan, China

**Correction: Veterinary Research (2025) 56:112** 10.1186/s13567-025-01550-8

Following publication of the original article [1], the authors identified that the captions for Figure 2 through Figure 9 were incorrectly included.

The figures were renumber as follows:

Figure 2 became Figure 9

Figure 3 became Figure 2

Figure 4 became Figure 3

Figure 5 became Figure 4

Figure 6 became Figure 5

Figure 7 became Figure 6

Figure 8 became Figure 7

Figure 9 became Figure 8

Figure 7 B legend has been updated:

From: **B** Left: Survival rate of ducklings infected with the RA-YM, RA-YM Δ*porV*, and RA-YM CΔ***porF*** strains. Right: Survival rates of ducklings infected with the RA-YM, RA-YM Δ*posF*, and RA-YM CΔ*posF* strains. Ten ten-day-old ducklings per group infected with 107 CFU of bacteria were assessed for survival. Statistical significance was determined using a log-rank (Mantel‒Cox) test.

To: From: **B** Left: Survival rate of ducklings infected with the RA-YM, RA-YM Δ*porV*, and RA-YM CΔ***porV*** strains. Right: Survival rates of ducklings infected with the RA-YM, RA-YM Δ*posF*, and RA-YM CΔ*posF* strains. Ten ten-day-old ducklings per group infected with 107 CFU of bacteria were assessed for survival. Statistical significance was determined using a log-rank (Mantel‒Cox) test.

The original article has been corrected.
