# Supplementary material for: PorV factor of the type IX secretion system and PosF porin act as adhesins in Riemerella anatipestifer infection
Source: Vet Res. 2025 Jun 8;56:112. doi: 10.1186/s13567-025-01550-8 (PMC12147357; doi:10.1186/s13567-025-01550-8)
Supplement: Supplementary file 1 — Additional file 1: The bacterial strains, plasmids and primers used in this study. [file 13567_2025_1550_MOESM1_ESM.docx]

**Additional file 1. Strains, primers and plasmids used in this study.**

| Strains, plasmids and primers | Description | Source |
| --- | --- | --- |
| Strains |  |  |
| RA-YM | *Riemerella anatipestifer* wild-type strain, serotype 1 | Laboratory preservation |
| RA-YM Δ*porV* | *porV* gene deletion mutant strain, Spec^R^ | This study |
| RA-YM CΔ*porV* | Complemented Δ*porV* strain, Spec^R^, Erm^R^ | This study |
| RA-YM Δ*posF* | *posF* gene deletion mutant strain, Spec^R^ | This study |
| RA-YM CΔ*posF* | Complemented Δ*posF* strain, Spec^R^, Erm^R^ | This study |
| *E. coli* DH5α | F- φ80 *lacZΔM15* Δ(*lacZYA-argF*) *U169* *endA1* *recA1* *hsdR17*(rk^-^, mk^+^) *supE44* λ^-^ *thi-1* *gyrA96 relA1 phoA* | Weidi, Shanghai, China |
| *E. coli* BL21 (DE3) | *E. coli* B F^-^ *dcm ompT* *hsdS* (rB‑ mB‑) *gal* [*malB*+] K-12 (λS) | Weidi, Shanghai, China |
| *E. coli* X7213 | *thi-1 thr-1 leuB6 glnV44 fhuA21 lacY1 recA1* RP4-2-Tc: Mu λ *pir1 asdA41* *zhf-2*:Tn*10* | Laboratory preservation |
| Plasmids  pMD18-T | TA cloning vector | TAKARA |
| pRE-112 | Suicide vector | Laboratory preservation |
| pRE-112-*porV*-LSR | Suicide vector | This study |
| pRE-112-*posF*-LSR | Suicide vector | This study |
| pRES-JX-Erm | Shuttle vector | Laboratory preservation |
| pRES-JX-Erm-*porV* | Shuttle vector | This study |
| pRES-JX-Erm-*posF* | Shuttle vector | This study |
| pET-16b | Expression vector | Novagen |
| pET-16b-CirA | Expression vector | This study |
| pET-16b-DnaK | Expression vector | This study |
| pET-16b-FadL | Expression vector | This study |
| pET-16b-RagB | Expression vector | This study |
| pET-16b-Lftp | Expression vector | This study |
| pET-16b-MotB | Expression vector | This study |
| pET-16b-PorV | Expression vector | This study |
| pET-16b-PosF | Expression vector | This study |
| pET-16b-TolC | Expression vector | This study |
| pET-16b-TR | Expression vector | This study |
| Primers (5’-3’) |  |  |
| CirA-F | CATATCGAAGGTCGTCATATGCAAACC  ACAGTCTATGCGTATGTG | This study |
| CirA-R | AGCCGGATCCTCGAGCATATGGAATCT  TACTTGTAGATTGATAAAATAGTTTCTA | This study |
| DnaK-F | CATATCGAAGGTCGTCATATGATGAGT  AAAATTATAGGAATTGACTTAGGTAC | This study |
| DnaK-R | AGCCGGATCCTCGAGCATATGTTTTAC  TTCTTCGAAATCTGCATCTT | This study |
| FadL-F | CATATCGAAGGTCGTCATATGCAGAAT  ATAGGTAATTCGCCCTATGC | This study |
| FadL-R | AGCCGGATCCTCGAGCATATGGTCGT  ACTGGCGTTTGTTAAACC | This study |
| RagB-F | CATATCGAAGGTCGTCATATGTGCGAA  AGATATTTAGATATAGTACCTACG | This study |
| RagB-R | AGCCGGATCCTCGAGCATATGCAATTC  TGGGTTCTTCAGCCTT | This study |
| Lftp-F | CATATCGAAGGTCGTCATATGGGAGGC  TTTAGAGTTTCTTTGCA | This study |
| Lftp-R | AGCCGGATCCTCGAGCATATGAAAAGG  GTTGTAAGATAGGCCTAAA | This study |
| MotB-F | CATATCGAAGGTCGTCATATGTGCGTCA  GCAAAAAGCAGTATG | This study |
| MotB-R | AGCCGGATCCTCGAGCATATGTCTTTTC  ACTGGAGCGATGTCC | This study |
| PorV-F | CATATCGAAGGTCGTCATATGCAGCAAA  AAGGGCAAGTACTTACG | This study |
| PorV-R | AGCCGGATCCTCGAGCATATGGCGGTCT  GCATTAGAAGTATCATC | This study |
| PosF-F | CATATCGAAGGTCGTCATATGAATGCATT  ACTTAACGCTAGTTCTCC | This study |
| PosF-R | AGCCGGATCCTCGAGCATATGAAACTGA  TAACCTAAACCTACTATAAAGAAACT | This study |
| TolC-F | CATATCGAAGGTCGTCATATGGCTCAGGAGTCAAGAATGTTGTCTC | This study |
| TolC-R | AGCCGGATCCTCGAGCATATGTTTTTCTGTTAAAGTTCTTAGTTTACCTTG | This study |
| TR-F | CATATCGAAGGTCGTCATATGAAAGATAGTATTAGTGTTAAACCAACAAATTT | This study |
| TR-R | AGCCGGATCCTCGAGCATATGAAGATTAAAATTGATGTAAACGGAATG | This study |
| PorV-Left arm-F | GATCCCAAGCTTCTTCTAGAGGTACCGGGTTCACCCAGATCATCTAAAAG | This study |
| PorV-Left arm-R | CGTTCCACTGATCTTTATTTTAATATTATCATTTTTTCTATTTG | This study |
| PorV-Spc-F | AAATAAAGATCAGTGGAACGAAAACTCACGTTAAG | This study |
| PorV-Spc-R | TTTAACCATACAGTAGTTTTAAAAGTAAGCACCTGTTA | This study |
| PorV-Right arm-F | AAAACTACTGTATGGTTAAAATAGATACAATTTCTTTA | This study |
| PorV-Right arm-R | TCCCGGGAGAGCTCGATATCGCATGCTTATGATAAGAATGACTCCAAAAATAAGC | This study |
| PosF-Left arm-F | GATCCCAAGCTTCTTCTAGAGGTACCTGAGAGTGACGGAGCAGATAGA | This study |
| PosF-Left arm-R | CGTTCCACTGAATGTTTTATTTTTTTATAAATTAG | This study |
| PosF-Spc-F | ATAAAACATTCAGTGGAACGAAAACTCACGTTA | This study |
| PosF-Spc-R | TAAAAAAGTACAGTAGTTTTAAAAGTAAGCACCTGTTA | This study |
| PosF-Right arm-F | AAAACTACTGTACTTTTTTAATAAGAAACAAAAAATACTCAG | This study |
| PosF-Right arm-R | TCCCGGGAGAGCTCGATATCGCATGCAATTAAAACTTTATTAGGAGCGAGTGTATTAA | This study |
| PorV-Promoter-F | TTGAGAACGATTTAGCATATGGCTATTACCGCAACTAACTAAAGCT | This study |
| PorV-Promoter-R | GTAATTTTCATAATGAATATTTCTAATATATTTAGAATAAATCTATTTC | This study |
| PorV-Complement-F | ATATTCATTATGAAAATTACAAAAAAACTACTTTTAGGA | This study |
| PorV- Complement -R | TATGCAGTTTGGGACCATATGTTAGCGGTCTGCATTAGAAGTATCA | This study |
| PosF- Promoter -F | TTGAGAACGATTTAGCATATGTTAACGACCAAACAGCACAGCA | This study |
| PosF- Promoter -R | CCTACGAATACTTTTTTCATTAAAAACCTATTCTTTTGAATATGTAAATTATAA | This study |
| PosF- Complement -F | ATGAAAAAAGTATTCGTAGGTTTAGCTAT | This study |
| PosF- Complement -R | TATGCAGTTTGGGACCATATGTTAAAACTGATAACCTAAACCTACTATAAAGAA | This study |

^R^Resistance.
