# Supplementary figures and images for: PorV factor of the type IX secretion system and PosF porin act as adhesins in Riemerella anatipestifer infection
Source: Vet Res. 2025 Jun 8;56:112. doi: 10.1186/s13567-025-01550-8 (PMC12147357; doi:10.1186/s13567-025-01550-8)

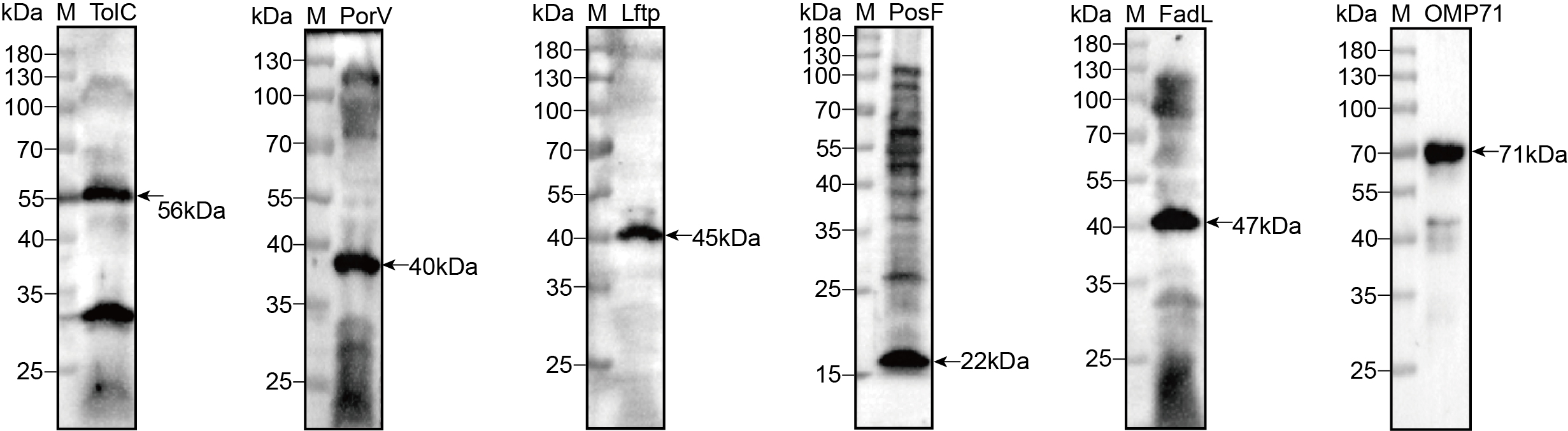

Supplement: Supplementary file 2 — Additional file 2: The specificity of each antibody was detected by western blotting. Substrate: Total OMPs of the R. anatipestifer RA-YM strain. The following antibodies were used: rabbit polyclonal antibodies against TolC, PorV, Lftp, PosF, FadL, and OMP71. [file 13567_2025_1550_MOESM2_ESM.jpg]

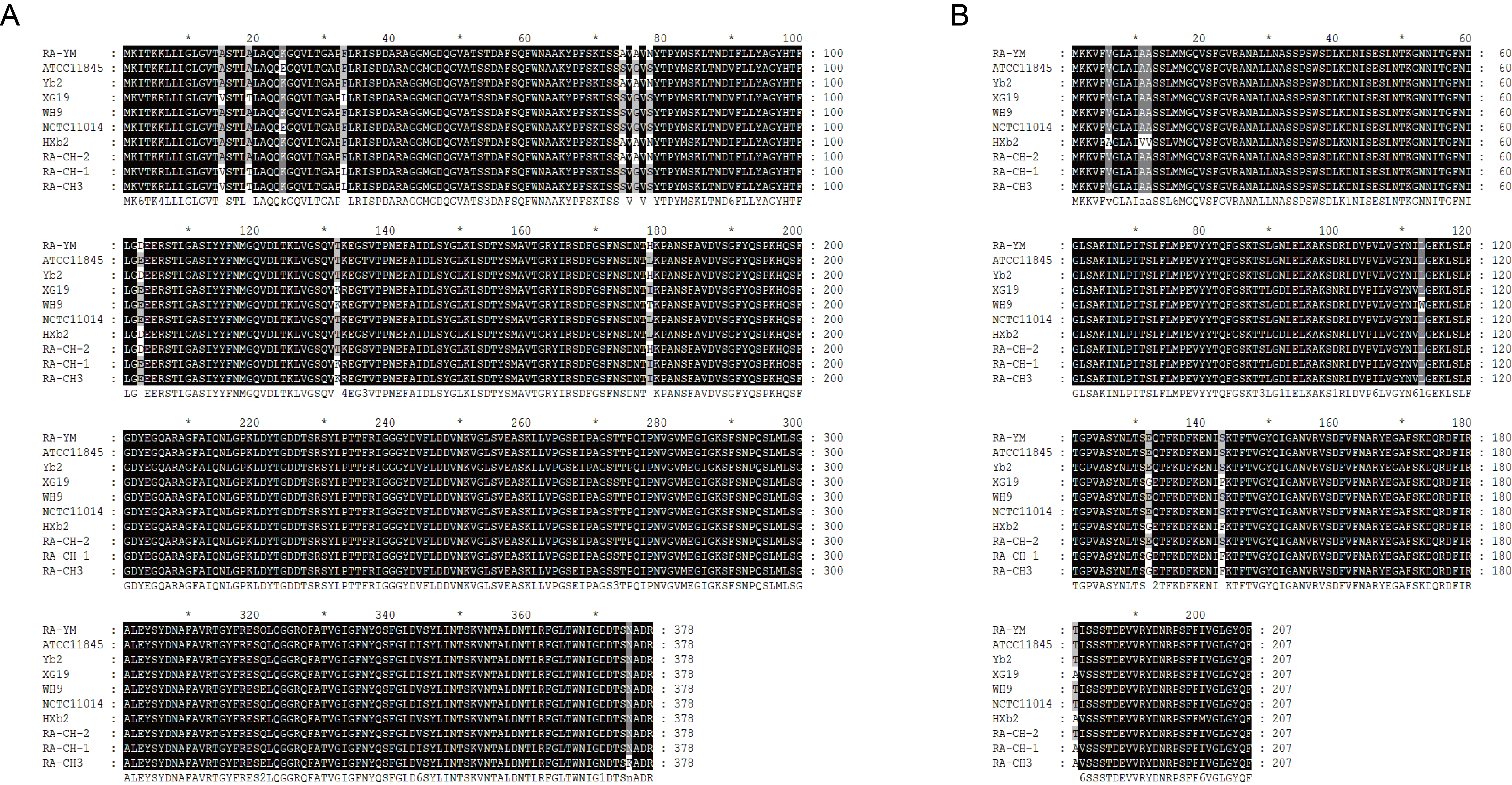

Supplement: Supplementary file 3 — Additional file 3: Conservation analysis of PorV and PosF proteins in different R. anatipestifer isolates. Conservation of PorV (A) and PosF (B) in different R. anatipestifer isolates analysed by MEGA and GENEDOC. [file 13567_2025_1550_MOESM3_ESM.jpg]

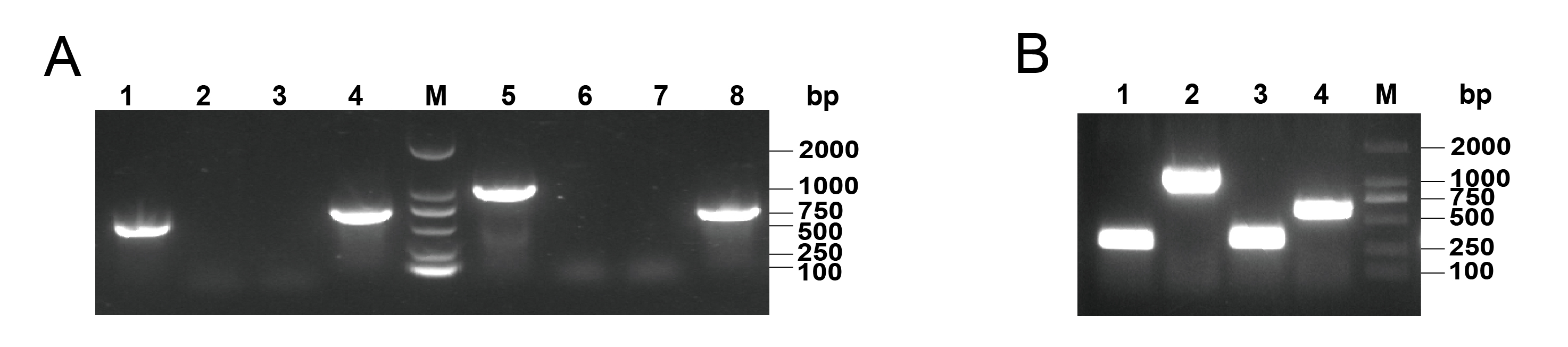

Supplement: Supplementary file 4 — Additional file 4: Construction of RA-YMΔporV, RA-YMCΔporV, RA-YMΔposF, and RA-YMCΔposF. (A) 1. Amplification of the posF gene (624 bp) in RA-YM. 2. Amplification of the posF gene in RA-YMΔposF. 3. Amplification of the spc gene without a promoter (726 bp) in RA-YM. 4. Amplification of the spc gene without a promoter in RA-YM. M: DL2000 DNA Marker. 5. Amplification of the porV gene (1137 bp) in RA-YM. 6. Amplification of the porV gene in RA-YMΔporV. 7. Amplification of the spc gene without a promoter in RA-YM. 8. Amplification of the spc gene without a promoter in RA-YMΔporV. (B) 1. Amplification of the promoter of the porV gene (335 bp). 2. Amplification of the porV gene (1137 bp). 3. Amplification of the promoter of the posF gene (344 bp). 4. Amplification of the posF gene (624 bp). M: DL2000 DNA Marker. [file 13567_2025_1550_MOESM4_ESM.jpg]
